# Microbial composition, functionality, and stress resilience or susceptibility: unraveling sex-specific patterns

**DOI:** 10.1186/s13293-024-00590-7

**Published:** 2024-02-26

**Authors:** Arax Tanelian, Bistra Nankova, Mariam Miari, Esther L. Sabban

**Affiliations:** 1 Department of Biochemistry and Molecular Biology, New York Medical College, Valhalla, NY 10595 USA; 2 Division of Newborn Medicine, Departments of Pediatrics, New York Medical College, Valhalla, NY 10595 USA; 3 Department of Psychiatry and Behavioral Science, New York Medical College, Valhalla, NY 10595 USA; 4 Department of Clinical Sciences in Malmo, Lund University Diabetes Center, Malmo, Sweden

**Keywords:** SPS, Sex differences, Anxiety, Resilience, Microbiome, SCFA

## Abstract

**Background:**

Following exposure to traumatic stress, women are twice as likely as men to develop mood disorders. Yet, individual responses to such stress vary, with some people developing stress-induced psychopathologies while others exhibit resilience. The factors influencing sex-related disparities in affective disorders as well as variations in resilience remain unclear; however, emerging evidence suggests differences in the gut microbiota play a role. In this study, using the single prolonged stress (SPS) model of post-traumatic stress disorder, we investigated pre- and post-existing differences in microbial composition, functionality, and metabolites that affect stress susceptibility or resilience in each sex.

**Methods:**

Male and female Sprague–Dawley rats were randomly assigned to control or SPS groups. Two weeks following SPS, the animals were exposed to a battery of behavioral tests and decapitated a day later. Based on their anxiety index, they were further categorized as SPS-resilient (SPS-R) or SPS-susceptible (SPS-S). On the day of dissection, cecum, and selected brain tissues were isolated. Stool samples were collected before and after SPS, whereas urine samples were taken before and 30 min into the SPS.

**Results:**

Before SPS exposure, the sympathoadrenal axis exhibited alterations within male subgroups only. Expression of tight junction protein claudin-5 was lower in brain of SPS-S males, but higher in SPS-R females following SPS. Across the study, alpha diversity remained consistently lower in males compared to females. Beta diversity revealed distinct separations between male and female susceptible groups before SPS, with this separation becoming evident in the resilient groups following SPS. At the genus level, Lactobacillus, Lachnospiraceae_Incertae_Sedis, and Barnesiella exhibited sex-specific alterations, displaying opposing abundances in each sex. Additionally, sex-specific changes were observed in microbial predictive functionality and targeted functional modules both before and after SPS. Alterations in the microbial short-chain fatty acids (SCFAs), were also observed, with major and minor SCFAs being lower in SPS-susceptible males whereas branched-chain SCFAs being higher in SPS-susceptible females.

**Conclusion:**

This study highlights distinct pre- and post-trauma differences in microbial composition, functionality, and metabolites, associated with stress resilience in male and female rats. The findings underscore the importance of developing sex-specific therapeutic strategies to effectively address stress-related disorders.

**Highlights**

- SPS model induces divergent anxiety and social behavioral responses to traumatic stress in both male and female rodents.
- SPS-resilient females displayed less anxiety-like behavior and initiated more interactions towards a juvenile rat than SPS-resilient males.
- Sex-specific pre-existing and SPS-induced differences in the gut microbial composition and predictive functionality were observed in susceptible and resilient rats.
- SPS-resilient males displayed elevated cecal acetate levels, whereas SPS-susceptible females exhibited heightened branched-chain SCFAs.

**Supplementary Information:**

The online version contains supplementary material available at 10.1186/s13293-024-00590-7.

## Plain language summary

After experiencing traumatic stress, women are more likely than men to develop mood disorders like anxiety and depression. However, people's responses to trauma vary—some develop mental health issues while others remain resilient. Recent research suggests that the bacteria in the gut might play a role in these differences. In this study, using a rat model of post-traumatic stress disorder (PTSD), we investigated whether there are differences in gut bacteria between male and female rats before and after stress exposure. The study involved two groups of rats—one not exposed to stress (control) and the other exposed to a traumatic event (stressed). The rats' behavior was evaluated using different tests to determine who among the males and females were vulnerable to stress and who were resilient. We found that even before the stress, there were differences in the types of bacteria and their functions in the guts of male and female rats. These differences were also linked to how they responded to stress. Interestingly, the bacteria that were more abundant in resilient males were found to be more abundant in vulnerable females. Additionally, the traumatic stress affected these bacteria and the substances they produce differently in males and females. In essence, our study demonstrates that the types of gut bacteria, their functions, and their products contribute to stress resilience in different ways for male and female rats. This insight suggests that tailored treatments specifically targeting these differences could be specially effective in treating stress-related issues.

**Supplementary Information:**

The online version contains supplementary material available at 10.1186/s13293-024-00590-7.

## Introduction

For decades, the field of psychiatry has largely focused on understanding mental health disorders through a gender-neutral lens, overlooking potential distinctions between males and females in terms of etiology, presentation, and treatment [1]. However, recent studies have shed light on the importance of considering sex differences in these conditions, as major neuropsychiatric disorders demonstrate imbalanced prevalence ratios between men and women. For instance, while neurodevelopmental disorders are more prevalent in men, mood disorders such as anxiety, depression, and post-traumatic stress disorder (PTSD) are more common in women [2–4]. Notably, following a traumatic event, women are twice as likely as men to develop PTSD, experience more chronic PTSD, and exhibit different symptoms and comorbidities than men [5–11]. While genetic, physiological, and environmental factors contribute to these differences, a growing body of evidence now underscores the significant impact sex differences in gut microbiota can have on the prevalence, symptomatology, and even in the biological underpinnings of various mental health disorders, as the elimination of microbiota diminishes several of the observed sex differences in disease outcomes [12–15].

The gut microbiota, a diverse community of microorganisms residing in the gastrointestinal tract, plays a crucial role in maintaining overall health and contributes to various physiological processes [16–18]. This complex ecosystem, in addition to actively participating in digestion, nutrient absorption, immune function, and metabolism, has been increasingly recognized for its bidirectional communication with the central nervous system (CNS), influencing brain development, function, and behavior [19–23]. This communication along the microbiota–gut–brain axis is sexually dimorphic and contributes to the development of distinct microbial communities, immune signaling pathways, and neuroinflammatory processes in males and females, ultimately giving rise to different mental health phenotypes [4, 24, 25].

Sex hormones may also play a role in the observed disparities in affective disorders between males and females, as these differences do not become apparent until puberty [26, 27]. Interestingly, sex dimorphism in the gut microbial composition also emerges after puberty [28, 29]. This indicates that sex hormones can manipulate the composition of the gut microbiota and contribute to the emergence of sex differences in microbial composition and the associated changes in the endocrine, immune, and neurotransmitter systems. However, the interaction between the sex hormones and gut microbiota appears to be reciprocal, resembling bidirectional communication of the gut–brain axis. For instance, administration of male cecal content to weaning female mice increases serum androstenedione and testosterone levels and shifts the microbial composition of the recipient female towards a male-based phenotype [29]. Additionally, the gut microbiota directly affects estrogen levels through deconjugation [30]. These findings collectively demonstrate that in addition to the bidirectional communication with the CNS, gut bacteria actively contribute to the regulation of sex hormone levels and their metabolism, potentially reinforcing or influencing the sex biases observed in neuropsychiatric disorders.

Although mood disorders, including PTSD, exhibit imbalanced prevalence ratios between the sexes, it is crucial to recognize that following a traumatic event, developing resilience (ability to adapt, cope, and recover from traumatic experiences) and susceptibility (developing long-term negative psychological and emotional consequences in response to traumatic events) are not confined to specific sex, but are unique to each individual [31, 32]. According to epidemiological studies, two-thirds of the population will be exposed to traumatic experience (s), but only 10% of men and 20% of women will develop PTSD [5, 6, 33, 34]. The underlying mechanisms that predispose individuals to resilience or susceptibility are unclear; however, clinical, and preclinical studies have suggested differences in gut microbial composition as a putative explanation for the observed phenotypic and epidemiological differences [35–38].

Previously, using single prolonged stress (SPS), an animal model of PTSD, we showed pre-existing and trauma-induced differences in the microbiota–gut–brain axis of SPS-resilient and SPS-susceptible male and female rats within each sex, which correlated with their ability to cope with the traumatic stress [39, 40]. In the current study, our focus shifts to a comparative analysis of sex differences in the gut microbial composition and functionality. Unlike our previous work, which examined each sex in isolation, we now investigate and compare the microbial profiles of male and female rats, both before and following exposure to SPS, that confer resilience or susceptibility to the traumatic stressor. Understanding the impact of sex-specific microbial composition and functionality may provide valuable insights into the underlying differences in susceptibility to mood disorders, fostering resilience, and developing personalized and effective therapeutic approaches.

## Materials and methods

### Animals

All animal experiments complied with the ARRIVE guidelines and NIH Guide for the Care and Use of Laboratory Animals. The study was approved by the Institutional Animal Care and Use Committee (IACUC) of New York Medical College. Male and female outbred Sprague–Dawley rats 6–7 weeks of age (150–170 g) were purchased from Charles River Laboratories (Wilmington, MA, USA). Throughout the experiment, the animals of the same sex were housed two per cage to avoid isolation stress and were maintained under a 12-h light/dark cycle at 23 ± 1 °C. Food and water were provided ad libitum.

### Experimental design

**Fig. 1:**
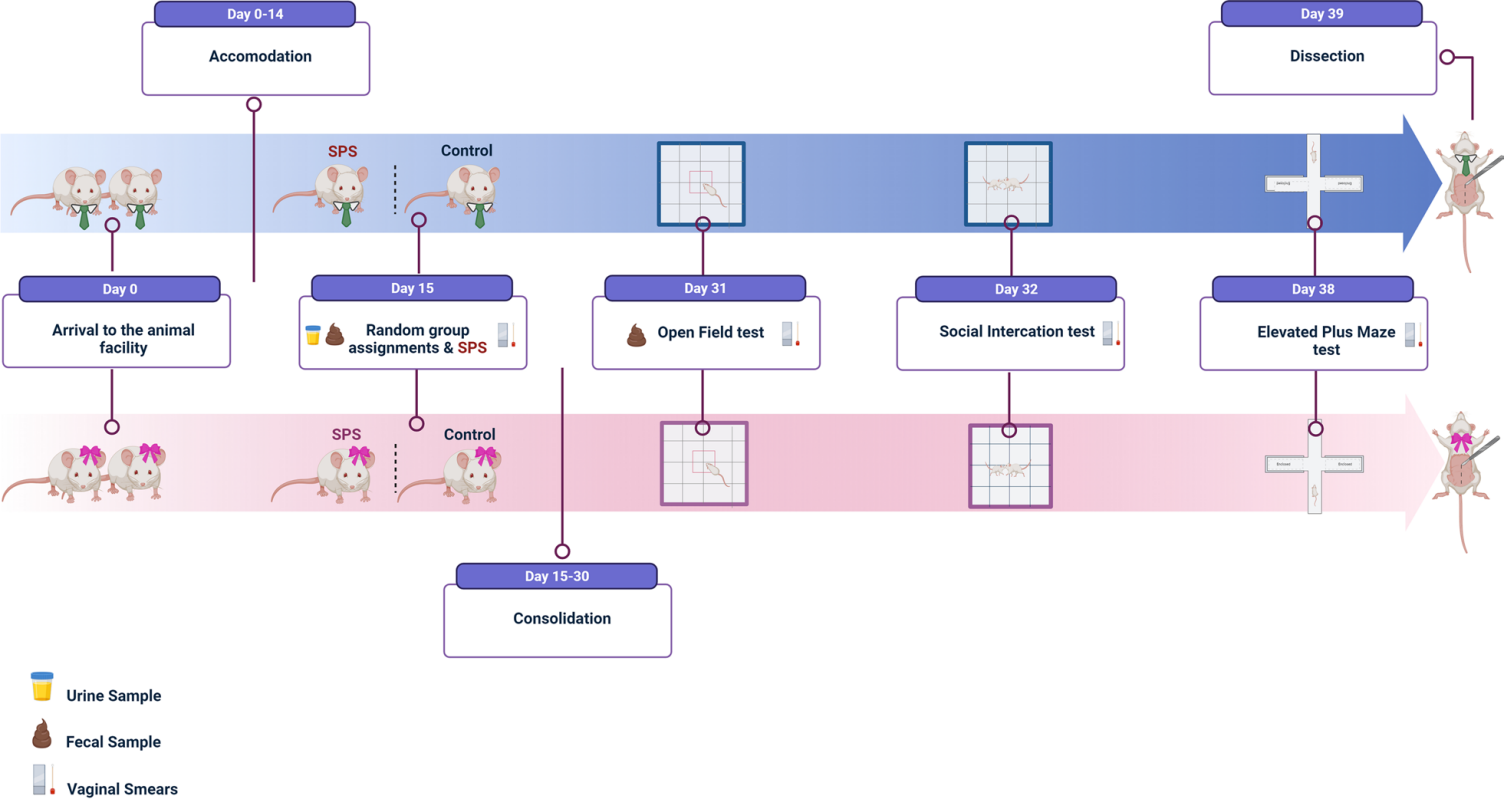
Experimental timeline. Two weeks following arrival, male and female rats were randomly divided into unstressed controls and experimental groups which got exposed to SPS. Two weeks later, a battery of behavioral tests was performed, and the animals were decapitated one day later. Stool samples were collected before SPS and on the day of open field test. Urine samples were collected before and during SPS. Vaginal smears were collected on the day of SPS and each behavioral test

Figure 1 illustrates the experimental timeline. After arriving at the animal facility, male and female rats underwent a 14-day acclimatization period. On day 15 the animals were randomly divided into unstressed control groups (*n* = 10/sex) or SPS experimental groups (*n* = 14/sex). The SPS experimental groups were exposed to the traumatic stress of SPS, while the controls were briefly handled. For seven days, the SPS-exposed groups remained undisturbed without bedding changes to consolidate the experience of traumatic stress, after which they were kept with normal bedding changes for the remainder of the experiment. Two weeks after SPS exposure, a series of behavioral tests were conducted in the order of Open Field (OF) on day 31, Social Interaction (SI) on day 32, and Elevated Plus Maze (EPM) on day 38. On day 39, one day after the last behavioral test, all animals were euthanized by decapitation. Additionally, the animals' weights were recorded on the day of SPS and two weeks after SPS (on the day of OF). Stool samples were collected before SPS and after the first behavioral test, urine samples were collected before SPS and 30 min into the immobilization step of SPS, and vaginal smears were collected on the day of SPS (day 15) and after each behavioral test (days 31, 32, 38).

### Single prolonged stress (SPS)

SPS, a widely used model for PTSD, elicits a strong stress response through psychological, physiological, and pharmacological pathways, inducing behavioral, neurobiological, and neuro-immune impairments [39–42]. A slightly modified version of SPS was performed as previously described [39, 43, 44]. Briefly, the animals were restrained by taping their limbs with surgical tape onto a custom-made metal board that restricted the motion of their heads. Immediately after 2 h of immobilization, the animals were subjected to a 20-min forced swim in a plexiglass cylinder filled two-thirds with fresh water at 24 °C. They were then dried and allowed to recuperate for 15 min, after which they were exposed to ethyl ether in a glass desiccator chamber until they lost consciousness.

### Behavioral tests

All behavioral tests were conducted in a dimly lit room. The tests were performed between 10 a.m. and 3 p.m. for males, and between 8 a.m. and 12:30 p.m. for females, to avoid the proestrus phase of the estrous cycle. All behavioral tests were videotaped using a ceiling camera and analyzed by trained individuals who were blinded to the experimental groups. To minimize potential carryover effects from prior behavioral assessments, the tests were administered in the order of least to most stressful. Before each test, the animals were given 30 min to acclimate to the testing room.

### Open field (OF)

Individual rats were placed in an open arena (40 × 32 × 24 cm, L × W × H) and were allowed to explore the arena for 5 min, after which they were returned to their respective home cages. Duration and number of entries into the virtual center zone (defined as 50% away from the edges), as well as number of rears were analyzed [39, 45].

### Social interaction (SI)

One day prior to the test, the animals were allowed to explore the open field arena for five mins to reduce the anxiety component in a novel environment. On the testing day, the animals were left to explore the field for 2 min after which a juvenile rat (50–75 g) of the same sex was introduced into the center of the arena. The animals were allowed to interact for 5 min, and their behavior was recorded. The time spent interacting and the number of approaches initiated by the test rats were scored and analyzed. The time spent in nose-to-nose sniffing, nose-to-anogenital sniffing, following, crawling over and under each other with physical contact, chasing, mounting, and wrestling initiated by the test rat was considered as the time spent engaged in social interaction [39, 40, 46].

### Elevated plus maze (EPM)

The apparatus (Stoelting, Wood Dale, IL, USA), 50 cm above ground level, has four cross-shaped platforms; two platforms with a 2-cm-high plexiglass fence wall are open, while the other two platforms with 40-cm-high opaque walls on the sides are closed. Arms of the same type are located opposite to each other. Each rat was placed on the central platform with its head towards an open arm and allowed 5 min to explore the maze [39, 40, 47]. The number of entries and time spent in each arm were scored and analyzed. Arm entry was defined as the entry of an arm with all four paws. The percentage of entries was calculated as the percentage of total open or closed arm entries to the total number of arm entries, and the time in the arms was calculated as a percentage of the total time of the test. The anxiety index was calculated as 1 − [(time spent in open arm/total time on the maze)/2 + (number of entries into the open arms/total exploration on the maze)/2] [48].

### Vaginal smears

Vaginal smears were collected from female rats, as previously described [40, 49] to determine the stage of ovulation. Briefly, the smears were collected using sterile swabs and distilled water and were left to dry on glass slides. The slides were then stained with 0.1% crystal violet and observed under a microscope at 10 × and 40 × magnifications. Views from distinct sections of the smear were analyzed by investigators blinded to the groups. Analysis of vaginal smears revealed that the vast majority of the animals were on estrus. All the animals were included in the analyses regardless of their ovulation stages (Additional file 1: Fig. S1A–D).

### Urine collection for epinephrine quantification

Urine samples were collected from each rat before SPS (baseline, rats were placed on pads for 20 min and were left to urinate voluntarily), and 30 min into immobilization (disposable dishes were placed under each rat). The samples were acidified immediately by addition of an equal volume of 0.01 M HCl and stored at − 80 °C for further analysis. Urine epinephrine levels were quantified using a commercially available competitive enzyme immunoassay kit (Rocky Mountain Diagnostics, Colorado Springs, CO) and normalized to urinary creatinine (DetectX Urinary Creatinine Kit, Arbor Assays, Ann Arbor, MI) concentrations in the same samples [39]. Epinephrine levels were assessed in urine based on the non-invasive nature of urine collection compared to the more intrusive blood collection method. This choice minimizes potential confounding factors and ensures a more accurate reflection of the physiological markers associated with SPS in our rats. Additionally, since epinephrine levels rise rapidly in blood, they can only be assessed in cannulated animals, which further supported our decision to utilize urine for a less intrusive and stress-free assessment.

### Tissue collection

Brains were dissected using a brain matrix. For ventral hippocampus (vHipp) sections, − 4.80 mm to − 5.20 mm to bregma were dissected and for medial prefrontal cortex (mPFC) sections, 1.5 mm to − 3.7 mm to bregma were isolated, flash frozen in liquid nitrogen and stored at − 80 °C until further use.

### Cecum and cecal SCFA quantification

Ceca were isolated, weighed and snapped frozen in liquid nitrogen and stored at − 80 °C until further use. The cecal weight was normalized to the body weight measured on the day of dissection. Cecal samples were sent to Gnotobiotics, Microbiology and Metagenomics Center (Boston, MA, USA) for SCFA analysis as previously described [39, 40].

### Western blot

Individual samples from vHipp and mPFC were homogenized in RIPA buffer. Protein concentration was determined by DC Protein Assay (Bio-Rad, Hercules, CA) with Bio-Tek plate reader, and 50 μg of total protein were separated on 4–10% gel and transferred to PVDF membranes (Bio-Rad). The membranes were blocked with 5% milk for 1 h at room temperature and incubated with primary anti-claudin-5 monoclonal antibody (1:500 dilution, Invitrogen Cat # 4C3C2) overnight at 4 °C. GAPDH (1:10,000, Cell Signaling, Cat # 14C10) was used as an internal control. After incubation with secondary antibody (IRDye 800CW) the bands were visualized using the Odyssey Infrared Imaging System (Li-Cor Biosciences, Lincoln, NB) and analyzed using ImageJ.

### Fecal microbiota sequencing

To determine the microbiome profile of the cohorts, fecal samples were collected aseptically from individual rats at the indicated time points (Fig. 1) between 10 a.m. and 2 p.m. to limit circadian influences on the microbiome and were stored at − 80 °C until further use. Briefly, prior to SPS, each animal was placed in a sterile cage for up to 15 min to defecate voluntarily. Upon defecation, the pellets were collected immediately into sterile tubes using sterile forceps and placed on dry ice. Post SPS, stool pellets were collected using sterile forceps while weighing the animals. Total DNA was extracted from each stool sample using a DNeasy PowerSoil Pro Kit (Qiagen, cat # 47014) according to the manufacturer's protocol. The extracted DNA was subjected to 16S V3–V4 rDNA sequencing and analysis at Psomagen (Rockville, MD, USA) as previously described [39, 40]. The 16S sequencing data are deposited to NCBI SRA, accession # PRJNA819002 (males) # PRJNA912323 (females).

### Microbial predictive functionality

For metagenomic function prediction, phylogenetic investigation of communities by reconstruction of unobserved states (PICRUSt) was used to infer KEGG pathways. PICRUSt infers the functional profiles of microbial communities based on their taxonomic composition. It predicts which functional genes are likely present in the microbial community, providing insights into the potential metabolic pathways and biological functions of the microbes in the sample.

### Data analysis

Statistical analyses were performed using the GraphPad Prism 9 software. Comparison of more than two groups was performed by one-way ANOVA for Gaussian distributions, whereas Kruskal–Wallis test was used for non-Gaussian distributions. Comparisons for more than two variables were done using two-way ANOVA. To compare group means from different time points, two-way repeated measures, or mixed-model ANOVA were used. Microbiome data were centered log-ratio (CLR) transformed using a composition library [50, 51]. Principal component analysis for beta diversity was performed in R (4.1.2) using the Aitchison distance as a distance matrix. Statistical significance was set at an α level of less than 0.05 (two-tailed). To correct for multiple testing, the Benjamini–Krieger–Yekutieli post hoc test was used with a q-value of 0.05 as a cut-off in all AVOVA tests. Effect sizes were calculated as partial eta square (*η*^2^). Data ≥ 2SD away from the mean was removed from microbiome analysis. Data are expressed as the mean ± SEM.

## Results

### Male and female rats exposed to SPS displayed sex-specific anxiety-like behavior and social impairments

To assess the animals’ anxiety/avoidance-like behavior, elevated plus maze (EPM) and open field (OF) tests were employed.

**Fig. 2:**
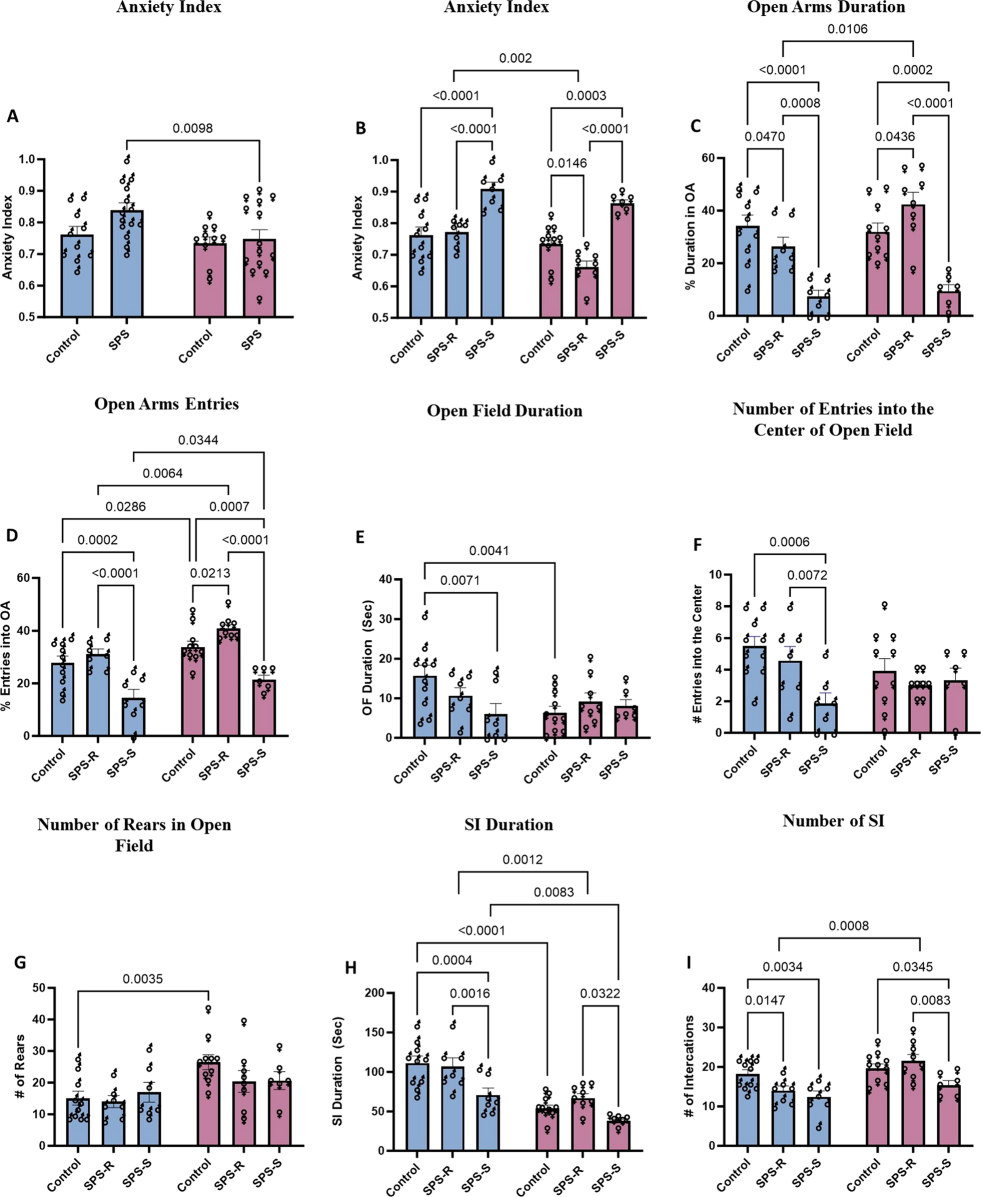
Sex differences in anxiety-like behavior and social impairments following SPS. Anxiety-like behavior tested on EPM:** A** Anxiety Index before group subdivisions, **B** Anxiety Index following group subdivisions, **C** % duration in open arms (OA), **D** % entries into open arms (OA). Anxiety-like behavior tested on OF: **E** duration in the center of the arena, **F** number of entries into the center of the arena, **G** number of rears. Social behavior tested on SI: **H** time spent interacting with a juvenile rat of same sex, **I** number of interactions initiated by the test rat towards the juvenile rat. Each symbol represents the value for an individual animal (blue bars—males: control *n* = 10, SPS-R *n* = 7, SPS-S *n* = 7; pink bars—females: control *n* = 10, SPS-R *n* = 8, SPS-S *n* = 6)

*On EPM*: A comparison between the male and female anxiety index revealed a significant sex effect (*F*_(1,44)_ = 5.2, *p* = 0.0275, *η*^2^ = 0.1050). SPS males displayed a significantly higher anxiety index than SPS females. No sex differences were observed in the anxiety indices of the unstressed controls (Fig. 2A). Irrespective of sex, animals in the SPS group showed divergent responses to the traumatic stress, with females displaying clearer separation (Fig. 2A). The anxiety index of the SPS treated males ranged from 0.7–1, whereas that of the females ranged from 0.5–0.9. Due to the observed variations in both sexes, animals in the SPS group with an anxiety index two standard deviations above the mean of their respective controls were subdivided into SPS-susceptible (SPS-S) subgroups, while the rest were grouped as SPS-resilient (SPS-R) [39, 40, 52, 53]. Following group subdivisions, significant group (*F*_(2,42)_ = 30.92, *p* < 0.0001, *η*^2^ = 0.5955) and sex (*F*_(1,42)_ = 12.03, *p* = 0.0012, *η*^2^ = 0.2220) effects were observed in the anxiety index with no interaction. Animals in the SPS-S subgroup of both sexes showed a significantly higher anxiety index than their respective controls and SPS-R subgroups (Fig. 2B). Interestingly, SPS-R females displayed a significantly lower anxiety index than SPS-R males (Fig. 2B). When the percentage duration in the open arms was calculated, a significant group effect (*F*_(2,42)_ = 27.48, *p* < 0.0001, *η*^2^ = 0.5667) and an interaction between sex and group (*F*_(2,42)_ = 3.410, *p* = 0.0424, *η*^2^ = 0.1392) were observed. Animals in the SPS-S subgroup, regardless of sex, spent significantly less time in the open arms of the maze compared to their respective controls and SPS-R subgroups. SPS-R females spent significantly more time in the open arms than SPS-R males (Fig. 2C). Additionally, the frequency of open arm entries showed significant group (*F*_(2,42)_ = 26.63, *p* < 0.0001, *η*^2^ = 0.5591) and sex (*F*_(1,42)_ = 14.7, *p* = 0.0004, *η*^2^ = 0.2592) effects, with no significant interaction. Male and female rats in the SPS-S subgroups displayed significantly fewer entries into the open arms than their respective controls and SPS-R subgroups. However, females showed more frequent entries into the open arms (OAs) than their respective male groups (Fig. 2D).

*On OF*: The time spent in the center of open field showed significant group and sex interaction (*F*_(2,42)_ = 3.567, *p* = 0.0371, *η*^2^ = 0.1446). Male rats in the SPS-S subgroup spent significantly less time in the center compared to their controls, while no differences were observed among females. Additionally, control males spent significantly more time in the center of the arena than control females (Fig. 2E). Regarding the frequency of entries into the center, a significant group effect was found (*F*_(2,42)_ = 4.482, *p* = 0.0172, *η*^2^ = 0.1757). SPS-S males displayed fewer entries into the center compared to control and SPS-R males. However, no differences were observed among females (Fig. 2F). An additional measure of anxiety-like behavior on the OF is rearing behavior [54]. The analysis of the number of rears on the open field demonstrated a significant group effect (*F*_(1,42)_ = 10.29, *p* = 0.0026, *η*^2^ = 0.1962), with female controls showing more rears compared to control males (Fig. 2G).

*On SI:* Social interaction (SI) test was used to evaluate the level of active interaction between the test animals of each subgroup and a novel juvenile rat of the same sex. Analysis of the time spent interaction revealed significant group (*F*_(2,42)_ = 9.513, *p* = 0.0004, *η*^2^ = 0.3115) and sex (*F*_(1,42)_ = 48.25, *p* < 0.0001, *η*^2^ = 0.5342) effects with no interaction. Males in the SPS-S subgroup spent less time interacting with the juvenile rat compared to their controls and SPS-R subgroup. In females, however, differences were only evident between SPS-S and SPS-R subgroups. When the time spent interacting was compared between the sexes, males, irrespective of the groups, spent more time interacting than the females (Fig. 2H). Similarly, the number of interactions showed significant group (*F*_(2,42)_ = 7.297, *p* = 0.0019, *η*^2^ = 0.2575) and sex (*F*_(1,42)_ = 12.78, *p* = 0.0009, *η*^2^ = 0.2336) effects with no interaction. Male rats in the SPS subgroup initiated significantly fewer approaches towards the juvenile rats compared to their controls, whereas SPS-S females displayed fewer interactions compared to both SPS-R subgroup and unstressed controls. Additionally, SPS-R females demonstrated more frequent social interactions than SPS-R males (Fig. 2I).

### Sex-specific differences in net weight gain following SPS exposure

**Fig. 3:**
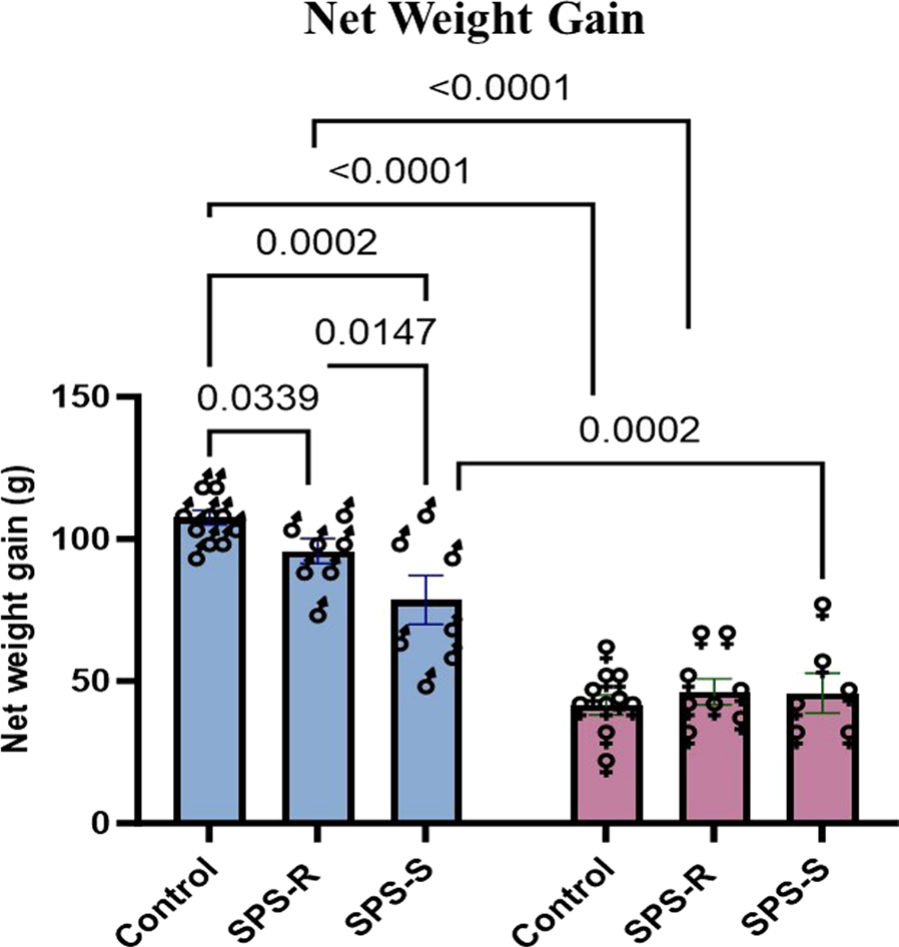
Sex differences in net weight gain. All animals were weighed on the day of SPS (day 15) and two weeks later (day 31), and the net weight gain was calculated. Each symbol represents the value for an individual animal (blue bars—males: control *n* = 10, SPS-R *n* = 7, SPS-S *n* = 7; pink bars—females: control *n* = 10, SPS-R *n* = 8, SPS-S *n* = 6)

As another measure of the stress response, animal body weight measurements were taken at the time of the SPS stressors, as well as 14 days afterward and the net weight gain from the day of the SPS stressors was calculated. While the group effect approached significance (*F*_(2,42)_ = 3.083, *p* = 0.0563, *η*^2^ = 0.1273), two-way ANOVA revealed significant sex effect (*F*_(1,42)_ = 142.1, *p* < 0.0001, *η*^2^ = 0.7711) and interaction between the two factors (*F*_(2,42)_ = 5.483, *p* = 0.0077, *η*^2^ = 0.2061). Male rats in SPS subgroups gained significantly less weight than their unstressed controls, however the net weight gain of the SPS-S subgroup was lower than that of the SPS-R subgroup. Moreover, males in all groups gained more weight than did the females. No group differences were observed among the females (Fig. 3).

### Sex-specific differences in adrenomedullary system of male and female rats before and after exposure to SPS

**Fig. 4:**
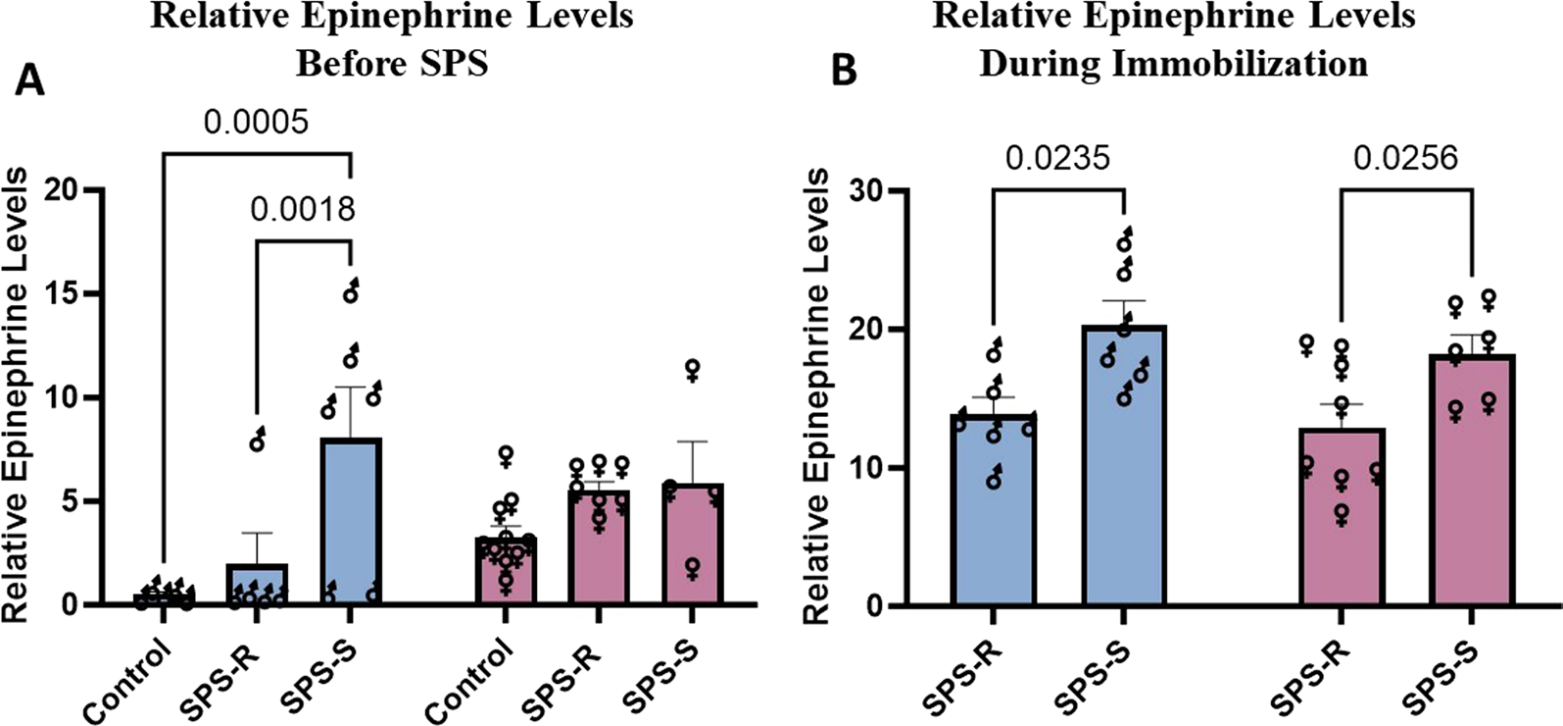
Sex differences in urinary epinephrine levels before SPS and 30 min into the immobilization step of SPS. Urine samples were collected before SPS and 30 min into immobilization step of SPS, to measure urinary epinephrine levels of individual animals. **A** Basal relative epinephrine levels, **B** relative epinephrine levels 30 min into the immobilization step of SPS. Each symbol represents the value for an individual animal. Due to technical difficulties, urine samples were not collected from every single animal. Moreover, during the immobilization step urine samples were not collected from the controls as they were not exposed to stress. Each symbol represents the value for an individual animal. Before SPS (blue bars—males: control *n* = 5, SPS-R *n* = 5, SPS-S *n* = 6; pink bars—females: control *n* = 10, SPS-R *n* = 7, SPS-S *n* = 4). Following SPS (blue bars—males: SPS-R *n* = 6, SPS-S *n* = 6, and pink bars—females: SPS-R *n* = 8, SPS-S *n* = 6)

To assess differences in the stress response of male and female rats, the urinary epinephrine levels were measured before and 30 min into the immobilization step of SPS. A comparison of the basal urinary epinephrine levels showed significant group effect (*F*_(2,31)_ = 7.212, *p* = 0.0027, *η*^2^ = 0.3173). Before exposure to SPS, male rats in the SPS-S subgroup displayed significantly higher levels of basal urinary epinephrine than their respective controls and the SPS-R subgroup. No differences were observed among the females (Fig. 4A). During the immobilization step of SPS, a significant group effect (*F*_(1,22)_ = 13.49, *p* = 0.013, *η*^2^ = 0.3792) was observed, with the urinary epinephrine levels being higher in the SPS-S subgroups of both sexes compared to their respective SPS-R subgroups (Fig. 4B).

### Sex-specific differences in the expression of tight junction protein in ventral hippocampus and medial prefrontal cortex following SPS exposure

**Fig. 5:**
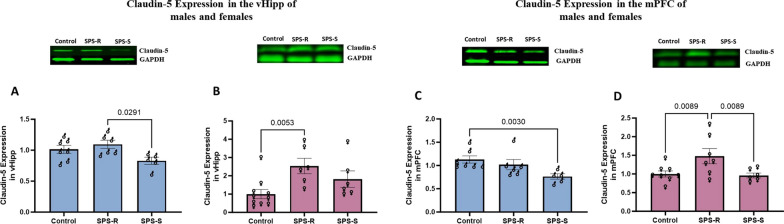
Sex-specific alterations in claudin-5 expression in the brain following SPS exposure. Ventral hippocampus (vHipp) and medial prefrontal cortex (mPFC) of each animal were dissected and Western blot was performed to analyze the expression of tight junction protein claudin-5. Expression of claudin-5 in **A** vHipp of males and **B** females, **C** mPFC of males and **D** females. Representative Western blots are shown. The images have been previously published in Neurobiology of Stress [39]. The analysis was performed using one-way ANOVA, as the samples from males and females were not run simultaneously. The values are normalized to their respective controls taken as 1. Claudin-5 protein expression data in the vHipp. of females, and in the mPFC of males were non-parametrically distributed and were analyzed using Kruskal–Wallis test followed by FDR corrected multiple comparison test. The rest of the data passed the normality test and were analyzed using one-way ANOVA, followed FDR corrected multiple comparison test. Each symbol represents the value for an individual animal. vHipp (blue bars—males control *n* = 7, SPS-R *n* = 6, SPS-S *n* = 5; pink bars—females control *n* = 10, SPS-R *n* = 6, SPS-S *n* = 6);mPFC (blue bars—males control *n* = 7, SPS-R *n* = 6, SPS-S *n* = 5; pink bars—females control *n* = 8, SPS-R *n* = 7, SPS-S *n* = 6)

Changes in blood–brain barrier (BBB) permeability are frequently reported in mood disorders [55]. We indirectly evaluated the permeability of the ventral hippocampus (vHipp) and medial prefrontal cortex (mPFC) by quantifying the levels of the tight junction protein claudin-5, known as the gatekeeper of neurological functions [56]. In males, the SPS-S subgroup exhibited significantly lower expression levels of claudin-5 compared to SPS-R subgroup in vHipp (*F*_(2,15)_ = 4.019, *p* = 0.04) and compared to unstressed controls in mPFC (*H*_(3,18)_ = 10.18, *p* = 0.0019), suggesting increased BBB permeability (Fig. 5A,5C). While in the females, the SPS-R group displayed higher expression of claudin-5 than that of the unstressed controls in the vHipp (*H*_(3,22)_ = 13.35, *p* = 9.480, *p* = 0.0044) and higher than that of both unstressed controls and SPS-S subgroup in mPFC (*F*_(2,18)_ = 4.667, *p* = 0.0233). Notably, claudin-5 levels in the SPS-S females were comparable to those observed in the controls for both regions (Fig. 5B, D).

### Pre-existing and trauma-induced differences in the gut microbial composition in male and female rats

#### Alpha diversity was higher in females than in males both before and after exposure to SPS

**Fig. 6:**
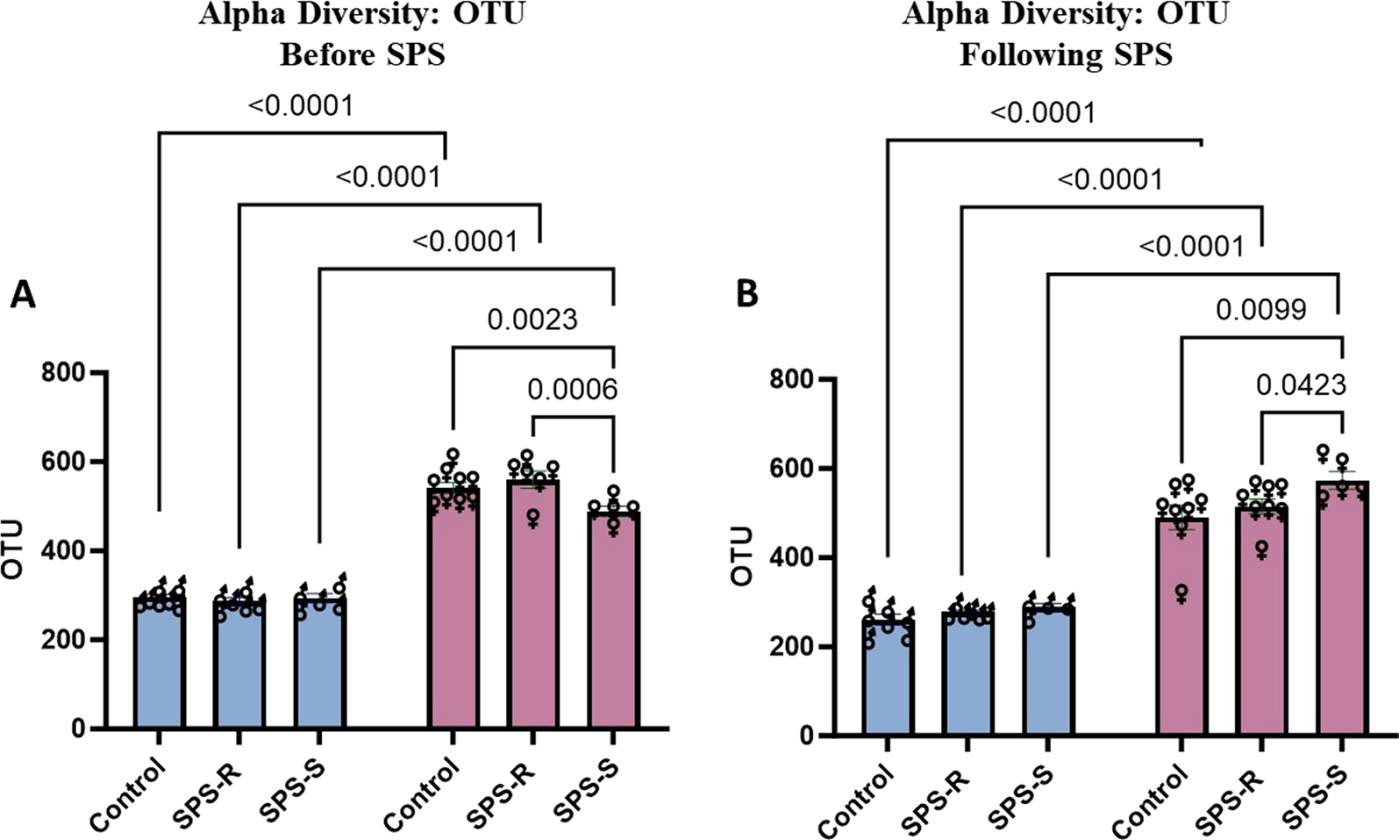
Sex differences in alpha diversity before and after SPS. Alpha diversity was measured using OTU counts **A** before SPS, **B** after SPS. Fecal samples collected from each rat were sent for 16S sequencing*.* Due to technical difficulties stool samples were not collected from every single animal. Each symbol represents the value for an individual animal. Before SPS (blue bars—males control *n* = 7, SPS-R *n* = 6, SPS-S *n* = 5; pink bars—females control *n* = 9, SPS-R *n* = 6, SPS-S *n* = 5), following SPS (blue bars—males control *n* = 7, SPS-R *n* = 6, SPS-S *n* = 4; pink bars—females control *n* = 8, SPS-R *n* = 8, SPS-S *n* = 5)

*Before exposure to SPS.* Analysis of alpha diversity using OTUs revealed significant effects of both group (*F*_(2,32)_ = 3.489, *p* = 0.0426, *η*^2^ = 0.1793) and sex (*F*_(1,32)_ = 551.2, *p* < 0.0001, *η*^2^ = 0.9459), with strong interaction between the two factors (*F*_(2,32)_ = 4.427, *p* = 0.0201, *η*^2^ = 0.2162). While no group differences were observed in the OTU numbers among males, SPS-S females exhibited a significantly lower number of OTUs compared to SPS-R and control females. When the numbers were compared between the sexes, females, regardless of their groups, had significantly higher OTU counts than males (Fig. 6A).

*Following SPS exposure*. Two-way ANOVA revealed significant sex (*F*_(1,32)_ = 241.9, *p* < 0.0001, *η*^2^ = 0.8835) and group (*F*_(2,32)_ = 3.740, *p* = 0.0347, *η*^2^ = 0.1895) effects in OTU counts. Similar to the findings of before SPS exposure, OTUs remained significantly higher in females compared to males across all three groups. However, a notable change was observed in females after SPS exposure. Contrary to the pre-SPS results, the SPS-S subgroup exhibited significantly higher OTU numbers compared to SPS-R and unstressed control groups (Fig. 6B).

#### Beta diversity showed distinct separations between the sexes before and after exposure to SPS

**Fig. 7:**
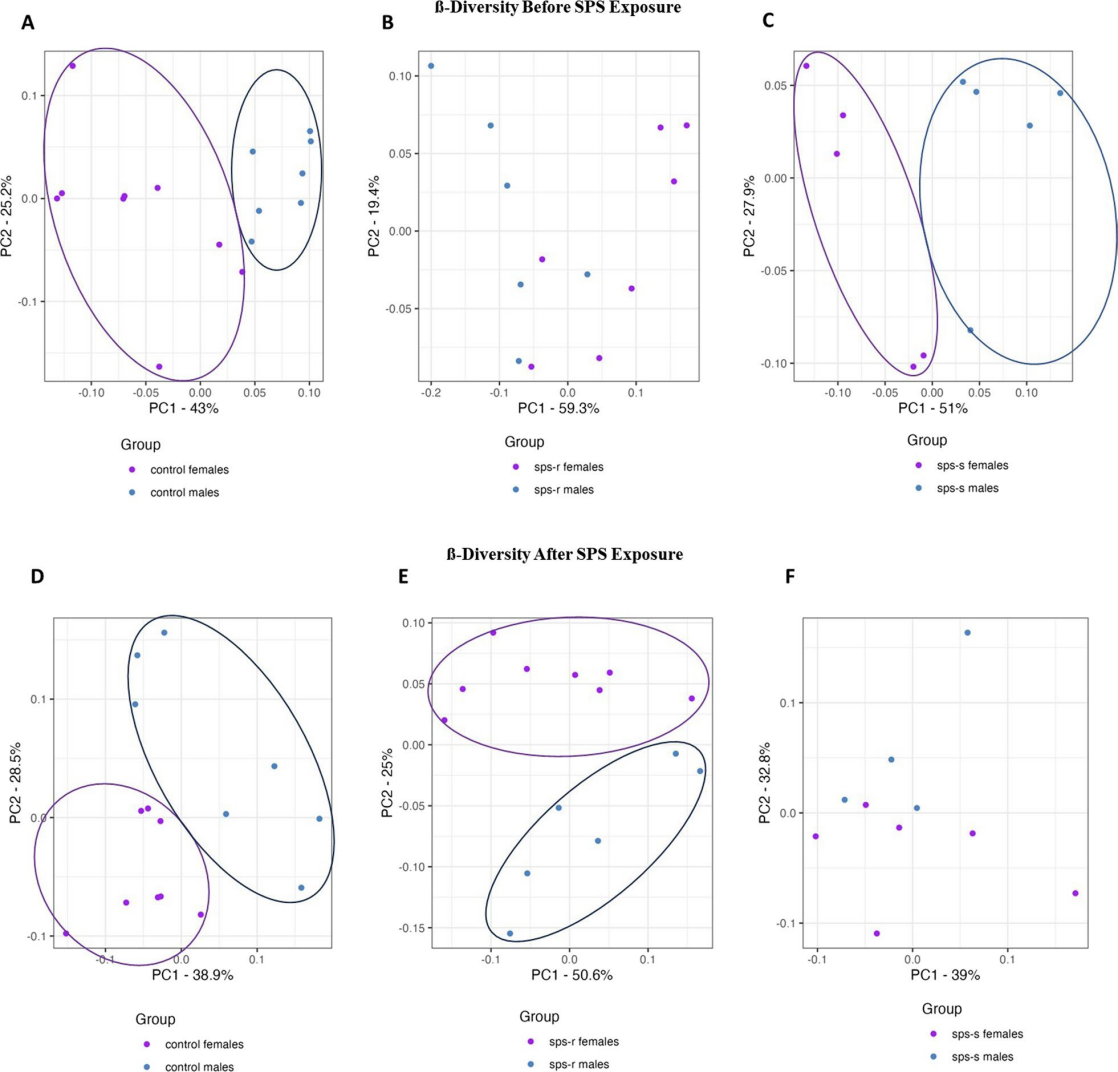
Sex differences in Beta diversity before and after SPS*.* Beta diversity was assessed using Aitchison distance. Before SPS **A** male vs female controls, **B** males vs females SPS-R subgroup, **C** males vs females SPS-S subgroup. After SPS: **D** male vs female controls, **E** males vs females SPS-R subgroup, **F** males vs females SPS-S subgroup. Due to technical difficulties stool samples were not collected from every single animal. Each symbol represents the value for an individual animal. Before SPS (blue bars—males control *n* = 7, SPS-R *n* = 6, SPS-S *n* = 5; pink bars—females control *n* = 9, SPS-R *n* = 7, SPS-S *n* = 5), following SPS (blue bars—males control *n* = 7, SPS-R *n* = 6, SPS-S *n* = 4; pink bars—females control *n* = 8, SPS-R *n* = 8, SPS-S *n* = 6)

*Before SPS exposure.* The Aitchison distance matrix, used as a measure of beta diversity [57], resulted in a PCA plot that displayed a distinct separation between male and female unstressed control groups (Fig. 7A), as well as between the SPS-S subgroups (Fig. 7C). Interestingly, there was no discernible separation observed between SPS-R males and females, indicating a similarity in microbial diversity in these two groups (Fig. 7B).

*Following SPS exposure.* A clear separation between control male and female rats persisted in the PCA plot after two weeks (Fig. 7D). However, unlike the pre-SPS results, the SPS-R male and female subgroups now exhibited a clear separation (Fig. 7E), whereas no distinct separation was observed within the SPS-S subgroups (Fig. 7F).

#### Sex-specific differences were observed at the genus level in males and females before and two weeks following SPS exposure

**Fig. 8:**
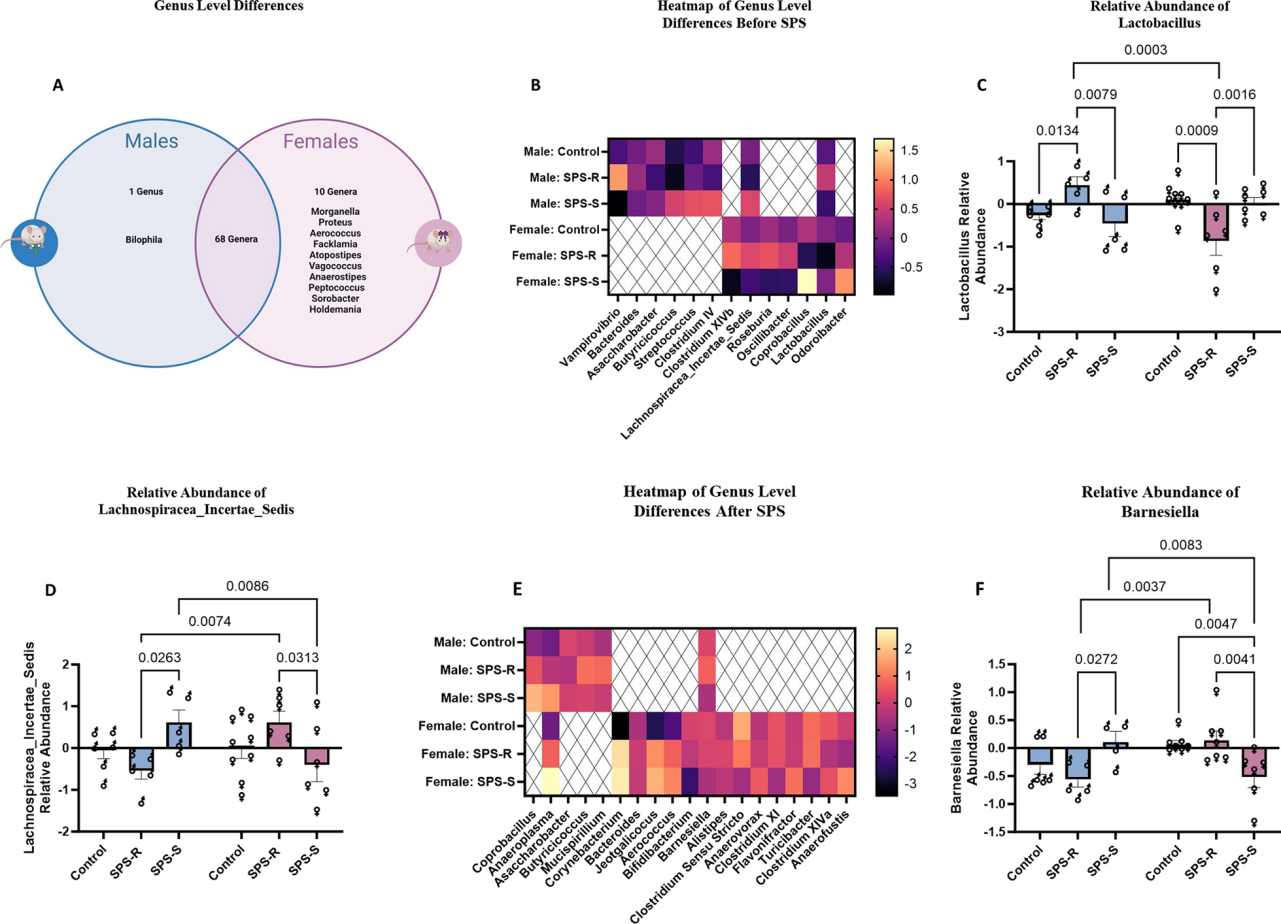
Sex differences in the gut microbial composition at genus level before and after SPS. **A** Venn diagram depicting shared and sex specific genera, **B** heat map of the genera which showed significant pre-existing group differences in each sex as well as in both sexes, **C** relative abundance of Lactobacillus, **D** relative abundance of Lachnospiraceae_Incertae_Sedis, **E** heat map of the genera which showed significant SPS-induced group differences in each sex and in both sexes, **F** relative abundance of Barnesiella. Due to technical difficulties stool samples were not collected from every single animal. Each symbol represents the value for an individual animal. Before SPS (blue bars—males control *n* = 6, SPS-R *n* = 5, SPS-S *n* = 5; pink bars—females control *n* = 9, SPS-R *n* = 6, SPS-S *n* = 7/6), following SPS (blue bars—males control *n* = 6, SPS-R *n* = 5, SPS-S *n* = 4; pink bars—females control *n* = 7, SPS-R *n* = 7, SPS-S *n* = 6)

Genus-level analysis of the gut microbial composition revealed that a total of 68 genera were shared between males and females, yet ten genera were exclusively present in females, and one genus was present only in males both before and after SPS exposure (Fig. 8A).

*Pre-existing differences:* Among these 68 shared genera, sex-specific group differences were observed in males and females (Fig. 8B). However, two genera, namely, Lactobacillus, and Lachnospiraceae_Incertae_Sedis, showed significant group differences in both sexes (Fig. 8B). The relative abundance of the genus Lactobacillus showed significant sex and group interaction (*F*_(2,32)_ = 11.52, *p* = 0.0002, *η*^2^ = 0.4182). In male rats, its abundance was significantly higher in the SPS-R subgroup than in the SPS-S subgroup, whereas in female rats, the abundance of Lactobacillus was significantly lower in the SPS-R subgroup than in the SPS-S subgroup and unstressed controls (Fig. 8C). When the abundance of Lactobacillus was compared between the sexes, female rats in the SPS-R subgroup showed significantly lower abundance than SPS-R males (Fig. 8C). Similarly, the relative abundance of the genus Lachnospiraceae_Incertae_Sedis showed a strong interaction between group and sex (*F*_(2,31)_ = 6.833, *p* = 0.0035, *η*^2^ = 0.3067). SPS-S males had significantly higher abundance of Lachnospiraceae_Incertae_Sedis compared to SPS-R males, whereas in females, the opposite was observed, with SPS-S subgroup showing significantly lower abundance than SPS-R subgroup (Fig. 8D). Moreover, when the abundance was compared between the sexes, SPS-R males showed significantly lower abundance of Lachnospiraceae_Incertae_Sedis than SPS-R females, whereas SPS-S males showed higher abundance than SPS-S females (Fig. 8D).

*Following SPS exposure.* To assess how gut microbial communities were altered after SPS, differences in gut microbial composition were evaluated. As before SPS, several sex-specific group differences were observed, with 2 genera showing alterations in both males and females (Fig. 8E). Yet only differences in the genus Barnesiella showed significant interaction between sex and group (*F*_(2,29)_ = 8.635, *p* = 0.0011, *η*^2^ = 0.3725). Following exposure to SPS, the abundance of genus Barnesiella was significantly higher in SPS-S males compared to SPS-R males, whereas in females it was significantly lower in SPS-S subgroup compared to SPS-R and unstressed controls (Fig. 8F). Comparison of the genus Barnesiella between the sexes, revealed higher abundance in SPS-R females than in SPS-R males, and significantly lower abundance in SPS-S females than in SPS-S males (Fig. 8F).

#### Sex differences in gut microbial predictive functionality before and after SPS

Next, the predictive functional profiles of the microbes were evaluated, as the link between microbial taxonomic composition and metabolic response is not direct [58]. Analyzing microbial functionality goes beyond understanding the composition of gut microbiota. It enables the comprehension of not only the potential biological activities and metabolic pathways, but also how the composition of the gut microbiota may influence or contribute to specific diseases and physiological processes.

**Fig. 9:**
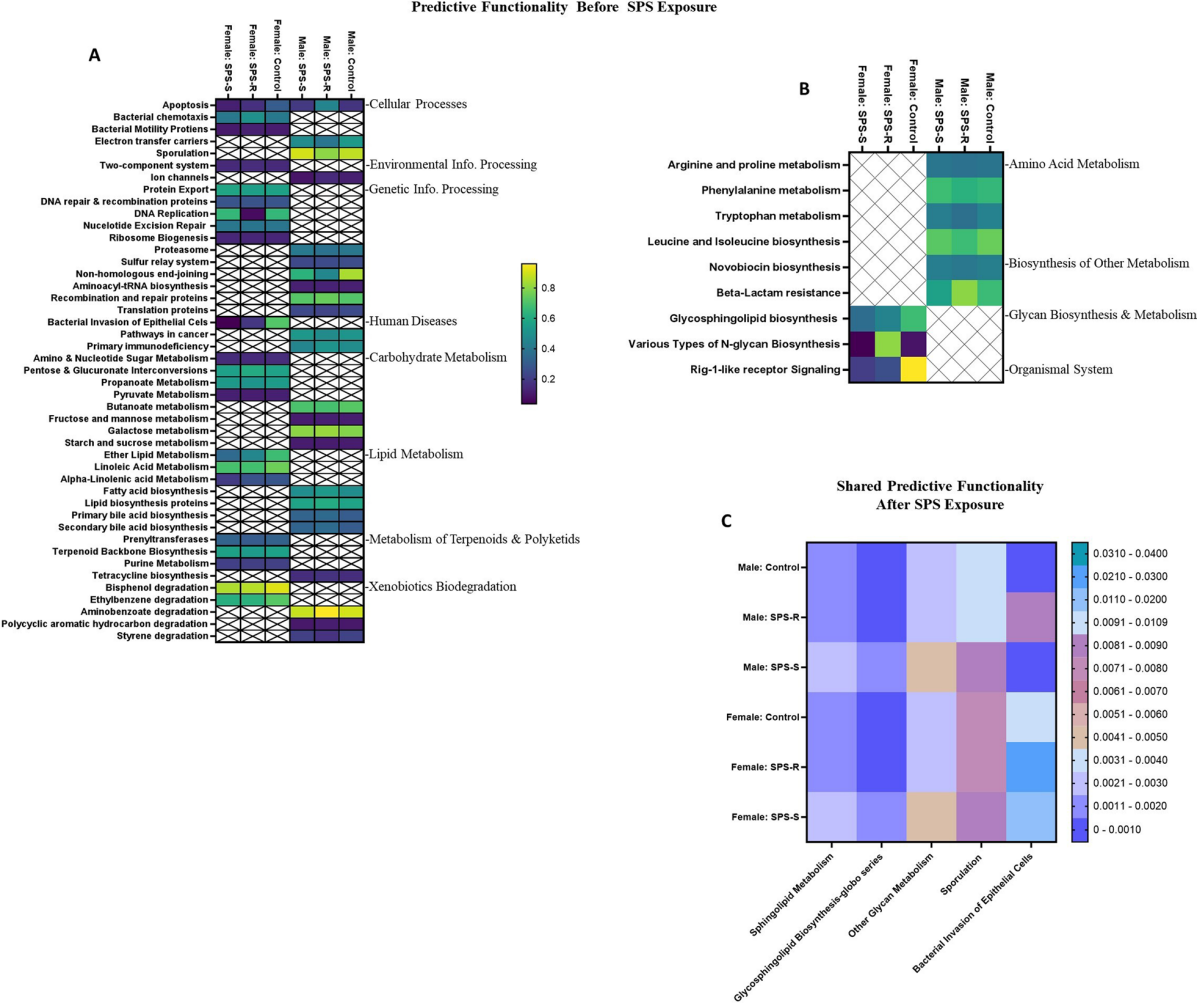
Sex differences in the gut microbial predictive functionality before and after SPS. **A** Heat map of the microbial predictive functionality which showed pre-existing group differences in both sexes (right side: general functional pathways, left side: specific domains in each pathway), **B** heat map of the microbial predictive functionality which showed pre-existing group differences in males or females only (right side: general functional pathways, left side: specific domains in each pathway), **C** heat map of the microbial predictive functionality which showed SPS-induced group differences in both males and females*.* Due to technical difficulties stool samples were not collected from every single animal. Each symbol represents the value for an individual animal. Before SPS (blue bars—males control *n* = 7, SPS-R *n* = 6, SPS-S *n* = 5; pink bars—females control *n* = 8, SPS-R *n* = 7, SPS-S *n* = 5), following SPS (blue bar—males control *n* = 7, SPS-R *n* = 6, SPS-S *n* = 4; pink bar—females control *n* = 7/8, SPS-R *n* = 7/8, SPS-S *n* = 5/6)

*Before SPS exposure.* Cellular processes, environmental and genetic information processing, human diseases, carbohydrate, lipid, terpenoid, and polyketide metabolism, and xenobiotic degradation showed significant alterations in the SPS-R and SPS-S subgroups in both sexes, although the pathways involved in each domain were distinct for males and females (Fig. 9A). On the other hand, pathways involved in amino acid metabolism and biosynthesis of other metabolites showed group differences only in males, whereas pathways involved in glycan biosynthesis and metabolism showed group differences only in females (Fig. 9B). The only common pathway between males and females was apoptosis in cellular processes, which showed a significant group effect (*F*_(2,32)_ = 3.560, *p* = 0.0402, *η*^2^ = 0.1822) and interaction between sex and group (*F*_(2,32)_ = 11.51, *p* = 0.0002, *η*^2^ = 0.4189). In males, the pathways involved in apoptosis were higher in SPS-R subgroup than in their controls and SPS-S subgroup, whereas in females, it was lower in both SPS subgroups than in the unstressed controls. Moreover, apoptosis was higher in SPS-R males than in SPS-R females (Additional file 1: Fig. S2A).

*Following SPS exposure.* Five pathways were found to be common between the sexes (Fig. 9C). Among these pathways, Sphingolipid metabolism (*F*_(2,32)_ = 11.08, *p* = 0.0002, *η*^2^ = 0.4098), and glycosphingolipid biosynthesis-globo series (*F*_(2,32)_ = 10.15, *p* = 0.0004, *η*^2^ = 0.3889) exhibited significant group differences only. On the other hand, other glycan metabolism showed both group (*F*_(2,32)_ = 17.06, *p* < 0.0001, *η*^2^ = 0.5162) and sex (*F*_(1,32)_ = 8.328, *p* = 0.0069, *η*^2^ = 0.2062) effects, with all three pathways being significantly higher in the SPS-S subgroup compared to SPS-R and unstressed controls, regardless of sex (Additional file 1: Fig. S2B–D). Sporulation demonstrated a significant sex effect (*F*_(1,32)_ = 15.82, *p* = 0.0004, *η*^2^ = 0.3182) with the pathway being higher in control and SPS-R males than in females (Additional file 1: Fig. S2E). Finally, bacterial invasion of epithelial cells exhibited significant sex (*F*_(1,32)_ = 43.58, *p* < 0.0001, *η*^2^ = 0.5760) and group (*F*_(2,32)_ = 4.668, *p* = 0.0166, *η*^2^ = 0.2254) effects, along with an interaction between the two factors (*F*_(2,32)_ = 4.802, *p* = 0.015, *η*^2^ = 0.2309). Irrespective of their groups, females showed strikingly higher levels of bacterial invasion than males. Notably, SPS-R females displayed higher levels of bacterial invasion compared to SPS-S and unstressed control females (Additional file 1: Fig. S2F).

### Sex-specific differences in gut–brain modules and gut–metabolic modules

To expand the functional analysis and the interaction between gut microbiota and the brain towards more targeted functional annotation frameworks, gut–brain modules (GBMs) and gut–metabolic modules (GMMs) were examined to assess the functional capabilities of gut microbes. These modules represent specific functional pathways that have been identified in the literature as being linked to gut–brain communication or microbiota metabolism, respectively [59, 60]. GBMs specifically target molecules that have the ability to traverse both the intestinal epithelium and the blood–brain barrier, indicating their potential role in influencing brain function. On the other hand, GMMs focus on gut-specific bacterial and archaic metabolic processes, with a particular emphasis on anaerobic fermentation, which plays a vital role in gut health and overall microbial function.

#### Gut–brain module before and after SPS exposure

**Fig. 10:**
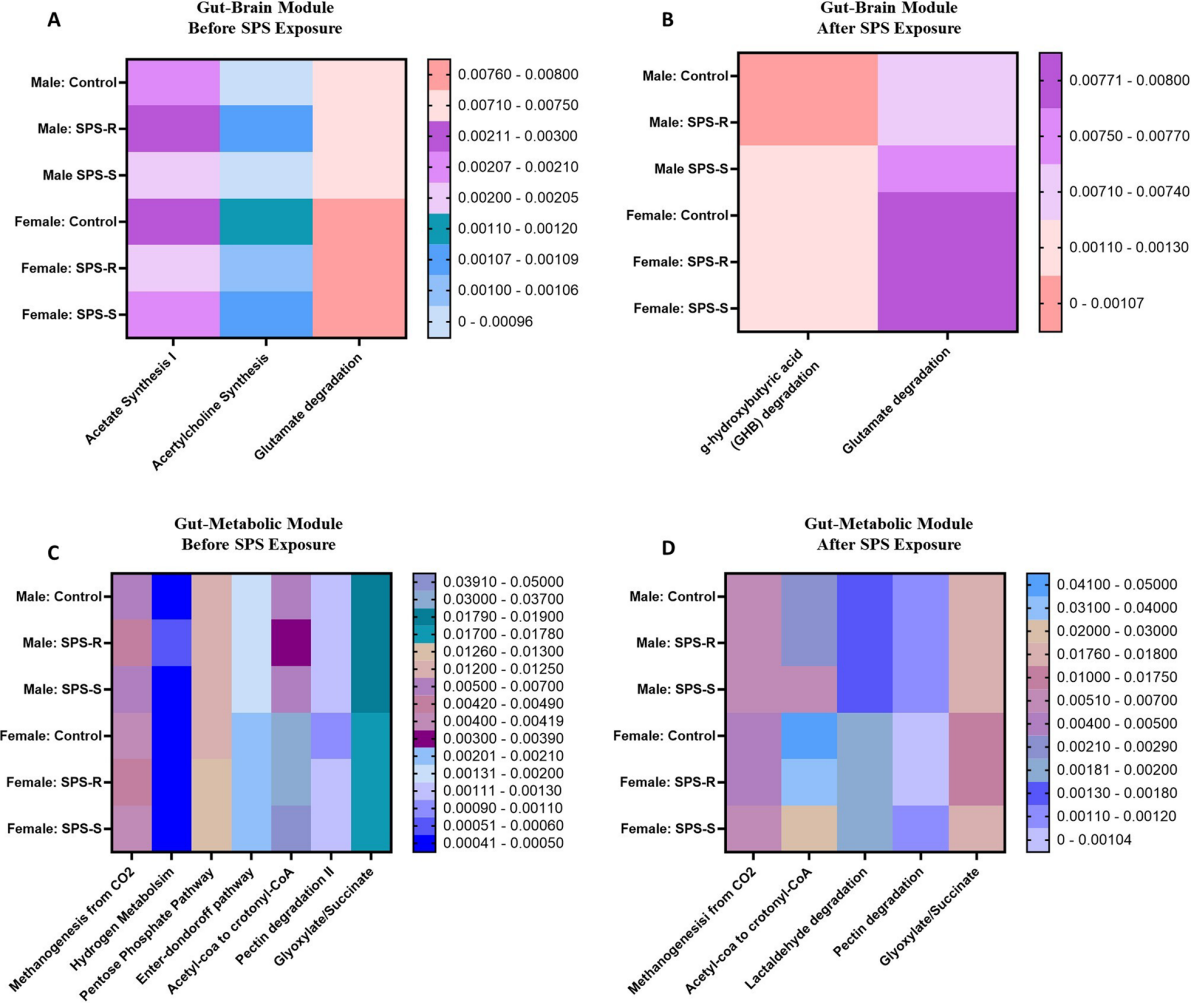
Sex differences in the gut–brain and gut–metabolic modules before and after SPS. **A** Heat map of the shared gut–brain module between males and females before SPS exposure, **B** heat map of the shared gut–brain module between males and females following SPS exposure, **C** heat map of the shared gut–metabolic module between males and females before SPS exposure, **D** heat map of the shared gut–metabolic module between males and females following SPS exposure*.* Due to technical difficulties stool samples were not collected from every single animal. Each symbol represents the value for an individual animal. Before SPS (blue bars—males control *n* = 6/7, SPS-R *n* = 5/6, SPS-S *n* = 5; pink bars—females control *n* = 8/9, SPS-R *n* = 6/7, SPS-S *n* = 5/6), following SPS (blue bars—males control *n* = 6/7, SPS-R *n* = 6, SPS-S *n* = 4; pink bars—females control *n* = 8, SPS-R *n* = 8, SPS-S *n* = 5/6). The values of acetyl-CoA to crotonyl-CoA were multiplied by 1000 to facilitate their integration into the heat map alongside the other data points

*Before exposure to SPS.* Three gut–brain modules were shared between the sexes (Fig. 10A). Acetate synthesis I showed significant interaction between group and sex (*F*_(2,32)_ = 6.198, *p* = 0.0053, *η*^2^ = 0.2791). Its levels were significantly higher in SPS-R males compared to unstressed controls and SPS-S males as well as SPS-R females (Additional file 1: Fig. S3A). Acetylcholine synthesis showed significant sex effect (*F*_(1,32)_ = 12.12, *p* = 0.0015, *η*^2^ = 0.2756), with interaction between sex and group (*F*_(2,32)_ = 5.378, *p* = 0.0097, *η*^2^ = 0.2513). The module was higher in SPS-R males compared to SPS-S and unstressed control males. While no group differences were seen among the females, acetylcholine synthesis was higher in controls and SPS-S females than in their respective groups in males (Additional file 1: Fig. S3B**)**. Similarly, glutamate degradation I showed significant sex effect (*F*_(1,32)_ = 24.97, *p* < 0.0001, *η*^2^ = 0.4385) and was significantly higher in females than in males, irrespective of the groups (Additional file 1: Fig. S3C).

*Following SPS exposure*. Only 2 gut–brain modules were altered (Fig. 10B). Gamma-hydroxybutyric acid (GHB) degradation showed significant sex effect (*F*_(1,32)_ = 27.42, *p* < 0.0001, *η*^2^ = 0.4604), and it was more significant in control and SPS-R females than in males of the same group (Additional file 1: Fig. S3D**)**. On the other hand, glutamate degradation I showed significant group (*F*_(2,32)_ = 3.693, *p* = 0.0361, *η*^2^ = 0.1871) and sex (*F*_(1,32)_ = 37.09, *p* < 0.0001, *η*^2^ = 0.5363) effects. The module was higher in SPS-S males compared to SPS-R and unstressed control males. However, compared to females, glutamate degradation was significantly lower in males than in females (Additional file 1: Fig. S3E).

#### Gut–metabolic module before and after SPS exposure

*Before exposure to SPS.* Seven GMM pathways were shared between the sexes (Fig. 10C). Methanogenesis from CO_2_ showed significant sex effect (*F*_(1,32)_ = 13.35, *p* = 0.0009, *η*^2^ = 0.2931) and interaction between sex and group (*F*_(2,32)_ = 3.459, *p* = 0.0437, *η*^2^ = 0.1785). While no sex-specific group differences were observed, unstressed controls and SPS-S females had significantly lower levels of methanogenesis than did their respective male rats (Additional file 1: Fig. S4A). Similarly, hydrogen metabolism showed significant sex effect (*F*_(1,32)_ = 9.239, *p* = 0.0047, *η*^2^ = 0.2245) with significant interaction between sex and group (*F*_(2,32)_ = 5.061, *p* = 0.0123, *η*^2^ = 0.2390). Hydrogen metabolism was lower in SPS-S males than in controls and SPS-R males; but was higher in SPS-R males than in SPS-R females (Additional file 1: Fig. S4B). Pentose phosphate pathway also showed sex (*F*_(1,32)_ = 21.05, *p* < 0.0001, *η*^2^ = 0.397), and group (*F*_(2,32)_ = 3.335, *p* = 0.0484, *η*^2^ = 0.173) effects, with an interaction between the two factors (*F*_(2,32)_ = 7.459, *p* = 0.0022, *η*^2^ = 0.3182). It was lower in SPS-R males but higher in SPS-R females compared to their respective SPS-R and control groups. Moreover, the pathway was higher in SPS-R females than in SPS-R males (Additional file 1: Fig. S4C). Additionally Entner–Doudoroff pathway (*F*_(1,32)_ = 22.97, *p* < 0.0001, *η*^2^ = 0.4185) (Additional file 1: Fig. S4D), acetyl-CoA to crotonyl-CoA (*F*_(1,32)_ = 67.55, *p* < 0.0001, *η*^2^ = 0.6783) (Additional file 1: Fig. S4E), and glyoxylate/succinate (*F*_(1,32)_ = 50.57, *p* < 0.0001, *η*^2^ = 0.6127) (Additional file 1: Fig. S4F) showed only sex effects and were all higher in females than in males, with acetyl-CoA to crotonyl-CoA showing the most marked differences between males and females. Finally, Pectin degradation II showed significant sex effect (*F*_(1,32)_ = 11.78, *p* = 0.0017, *η*^2^ = 0.2680) and interaction between sex and group (*F*_(2,32)_ = 4.371, *p* = 0.0210, *η*^2^ = 0.2144), and was significantly lower in females than in males (Additional file 1: Fig. S4G).

*Following exposure to SPS.* Five modules were shared between the sexes (Fig. 10D). Methanogenesis from CO_2_ (*F*_(1,32)_ = 7.403, *p* = 0.0104, *η*^2^ = 0.1885) (Additional file 1: Fig. S5A**)**, pectin degradation II (*F*_(1,32)_ = 39.20, *p* < 0.0001, *η*^2^ = 0.5503) (Additional file 1: Fig. S5B), Glyoxylate/Succinate (*F*_(1,32)_ = 30.54, *p* < 0.0001, *η*^2^ = 0.4886) (Additional file 1: Fig. S5C**)**, and acetyl-CoA to crotonyl-CoA (*F*_(1,32)_ = 60.65, *p* < 0.0001, *η*^2^ = 0.6544) (Additional file 1: Fig. S5D**)** were similar to before SPS, with the acetyl-CoA to crotonyl-CoA module remaining strikingly higher in females compared to males. However, lactaldehyde degradation (*F*_(1,32)_ = 6.940, *p* = 0.0129, *η*^2^ = 0.1785) (Additional file 1: Fig. S5E**)** showed sex effect and alterations only after SPS and was higher in SPS-R females than in SPS-R males.

### Sex differences in cecal bacterial metabolites (short-chain fatty acids) following SPS

**Fig. 11:**
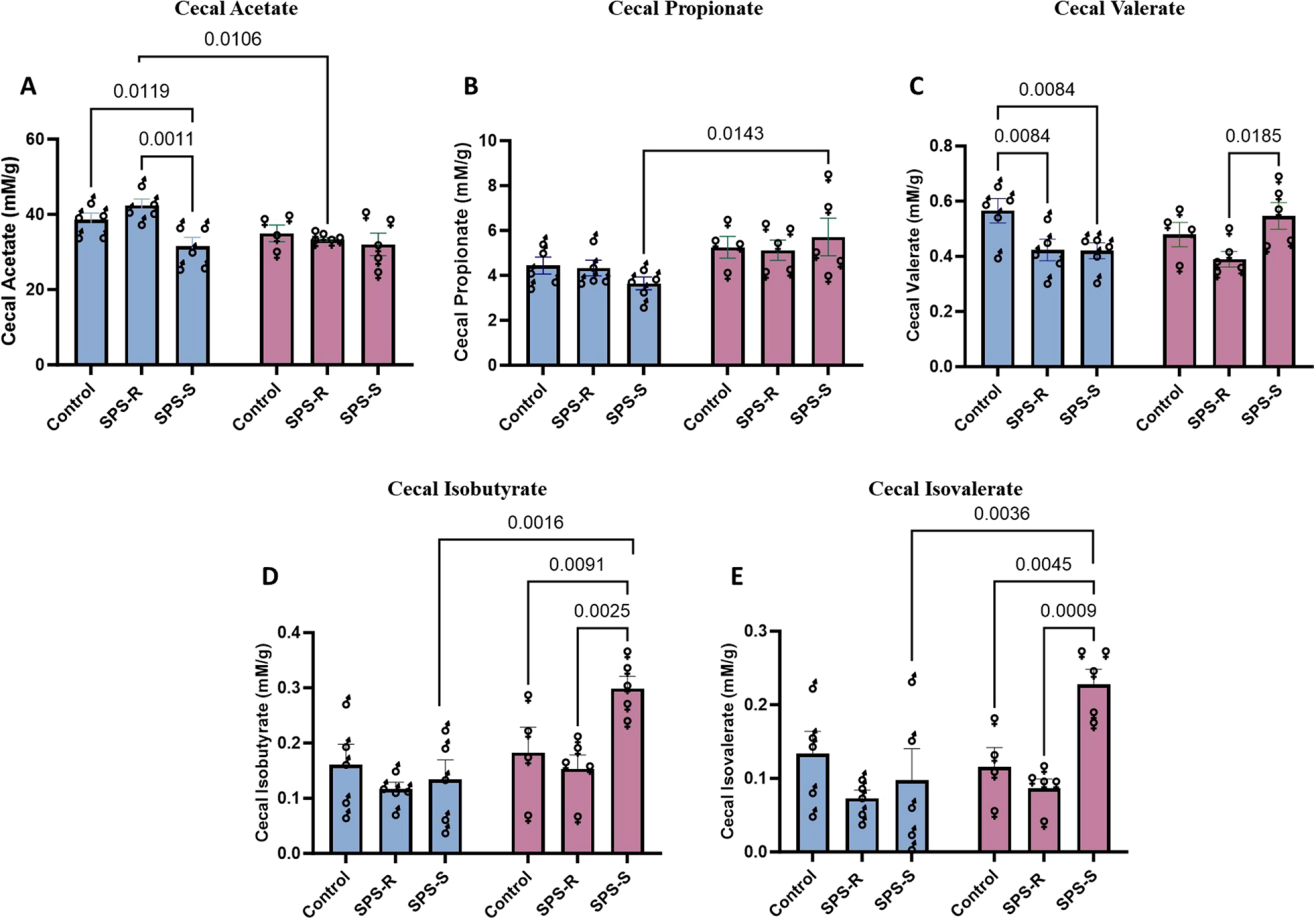
Sex differences in the gut microbial metabolites following SPS exposure. Cecum was collected from each rat on the day of dissection and randomly selected cecal samples from each subgroup were sent to SCFA analysis. **A** Levels of cecal acetate, **B** levels of cecal propionate, **C** levels of cecal valerate, **D** levels of cecal isobutyrate, **E** levels of cecal isovalerate. Each symbol represents the value for an individual animal (blue bars—males control *n* = 5, SPS-R *n* = 5, SPS-S *n* = 5; pink bars—females control *n* = 4, SPS-R *n* = 5, SPS-S *n* = 5)

The cecal weight at the time of dissection (day 39) was similar among the groups and between sexes (Additional file 1: Fig. S6A) however, the cecal SCFA levels were different. Cecal acetate levels showed significant group (*F*_(2,23)_ = 4.977, *p* = 0.016, *η*^2^ = 0.3021) and sex (*F*_(1,23)_ = 5.476, *p* = 0.0283, *η*^2^ = 0.1922) effects. In male rats, the levels of acetate were significantly lower in the SPS-S subgroup compared to SPS-R and unstressed controls; however, no differences were observed among females. When the levels were compared between males and females, SPS-R females showed significantly lower levels than did SPS-R males (Fig. 11A). The levels of propionate did not differ among the groups in males and females, yet significant sex effect (*F*_(1,23)_ = 8.936, *p* = 0.0066, *η*^2^ = 0.2808) was observed, with SPS-S females having significantly higher levels of propionate than SPS-S males (Fig. 11B). Although a sex effect (*F*_(1,23)_ = 5.904, *p* = 0.0233, *η*^2^ = 0.2042) was observed in the levels of cecal butyrate, multiple comparison tests did not show any differences within or between the sexes (Additional file 1: Fig. S6B).

In case of the minor SCFAs, the cecal levels of valerate showed significant group effect (*F*_(2,23)_ = 4.425, *p* = 0.0237, *η*^2^ = 0.2781), with a significant interaction between sex and group (*F*_(2,23)_ = 3.939, *p* = 0.0338, *η*^2^ = 0.2557). In males, valerate levels were similar between SPS-R and SPS-S subgroups and was significantly lower than the unstressed controls; whereas in females, the levels were significantly higher in SPS-S subgroup compared to SPS-R (Fig. 11C). As for caproate, two-way ANOVA analysis showed significant group effect (*F*_(2,23)_ = 4.710, *p* = 0.0193, *η*^2^ = 0.2909), yet no significance was observed with post hoc tests between or within sexes (Additional file 1: Fig. S6C).

Regarding the branched-chain fatty acids, the cecal levels of isobutyrate showed significant group (*F*_(2,23)_ = 3.655, *p* = 0.0418, *η*^2^ = 0.2411) and sex (*F*_(1,23)_ = 8.891, *p* = 0.0067, *η*^2^ = 0.2788) effect, with interaction between them approaching significance (*F*_(2,23)_ = 3.324, *p* = 0.054, *η*^2^ = 0.2242). In males no group differences were observed, yet in females, the SPS-S subgroup had higher levels compared to controls and SPS-R subgroup. When the levels were compared between the sexes, SPS-S females had significantly higher levels than SPS-S males (Fig. 11D). Finally, the levels of isovalerate showed significant group effect (*F*_(2,23)_ = 5.120, *p* = 0.0145, *η*^2^ = 0.3083) and interaction between sex and group (*F*_(2,23)_ = 4.36, *p* = 0.0248, *η*^2^ = 0.2745). Similar to isobutyrate levels, no group differences were found in males, yet in females, SPS-S subgroup had significantly higher levels compared to SPS-R and unstressed controls. Additionally, female SPS-S subgroup showed significantly higher levels of isovalerate than SPS-S males (Fig. 11E).

## Discussion

The current study demonstrated sex-specific differences in the behavioral, physiological, and microbial responses of susceptible and resilient rats following exposure to traumatic stress. Our results revealed, for the first time, both pre-existing and trauma-induced distinct variations in the adrenomedullary system (measured via urinary epinephrine), gut microbial composition (evaluated through alpha, beta-diversity and genus-level analysis), functionality (assessed through predictive pathways, GBMs, and GMMs), and metabolites (evaluated through SCFAs) and blood–brain barrier permeability (indicated by differences in claudin-5 expression) of resilient and susceptible male and female rats. These results shed new light on the intricate interplay between sex, stress response, and gut microbial dynamics, offering crucial insights into sex-specific reactions to stress.

Sex differences in the behavioral outcomes of male and female rats in response to chronic stress or SPS have been previously reported, however most of these studies have not assessed differences in resilient and susceptible animals [61–64]. This is of high importance, because more than two-thirds of the world population experience traumatic events at some point in their lives, and while most of these individuals (94.2%) are resilient and do not have long-term impairments, yet over 458 million people live with PTSD [65, 66]. Thus, having a stress model which captures interindividual variability in response to stress is crucial for understanding the mechanisms underlying these differing outcomes. The SPS model can induce divergent responses to traumatic stress in male and female rodents, with only a subset displaying anxiety-like behavior, whereas the remaining animals behave like unstressed controls [39, 40, 43, 67]. Recently, using machine learning, a study similarly subdivided the animals into SPS-R or SPS-S subgroups based on their anxiety measures on EPM and OF [68]. Here, we observed sex-specific differences in the behavioral phenotypes of susceptible and resilient rats following exposure to traumatic stress. Overall, the SPS-susceptible animals, regardless of sex, displayed similar behavioral responses on most of the parameters evaluated by the behavioral tests, whereas resilient females displayed similar or less behavioral impairments than resilient males. This observation, however, contradicts the usual trend seen in the general population, where women are more susceptible to mood disorders than men. One potential reason might be that the nature of responding to anxiety differs between males and females, with females possibly employing more active response strategies. For instance, on the EPM females displayed more frequent entries into the open arms, and on the SI test, they sought more frequent social contact. Another possibility is that the current tests used to measure anxiety-like behavior might not be suitable for accurately capturing sex differences [54, 69]. Some studies have suggested that increased anxiety-like behavior in females might become more evident, when they are exposed to specific, life-relevant conditions [70]. Thus, there is a need for careful selection of appropriate testing paradigms to better capture and understand the complexities of anxiety-related behaviors in both males and females.

Beyond observing sex differences in the behavioral phenotypes of susceptible and resilient rats, we also found differences in their physiological parameters. Males exhibited greater weight gain than females, regardless of their groups. While gonadal hormones likely contribute to these differences, recently, a new finding emerged concerning female POMC neurons within the hypothalamus. These neurons exhibit elevated expression of the Tap63 gene, which fosters accelerated firing rates and subsequently leads to diminished weight gain in females compared to males [71–73]. Similarly, sex differences in the adrenomedullary system were present. Basal epinephrine levels were higher in females than in males, although the difference was not statistically significant. Our findings align with previous studies indicating that females tend to exhibit a greater physiological response to handling, which could explain the initially higher epinephrine levels observed in female rats [74]. However, intriguingly, this effect seemed to dissipate during the immobilization step of the SPS procedure, as epinephrine levels became comparable between males and females. This could imply that the immobilization stressor evoked a similar response in both sexes.

Sex-specific responses to trauma, were also observed in the permeability of BBB in the animals exposed to SPS. While susceptible males displayed significantly lower levels of claudin-5 protein, compared to their unstressed controls and/or SPS-R subgroup, susceptible females demonstrated claudin-5 levels comparable to that of the unstressed controls. However, the levels of the tight junction protein were significantly higher in SPS-R subgroup than in unstressed controls and/or SPS-S subgroup. These observations suggest that (1) the impact of SPS varies between males and females, implying distinct underlying mechanisms, and (2) female resilience appears to rely on an active mechanism—elevated claudin-5 expression counteracts the effects of SPS—while male resilience may involve more passive strategies. Additionally, it is plausible that other tight junction proteins experienced downregulation in females following SPS, prompting the compensatory elevation of claudin-5. This underscores the need for a comprehensive exploration of other tight junction proteins in future studies.

The gut microbiota, through its bidirectional communication with the central nervous system, can significantly influence subjects' behavior [75, 76]. This communication is sexually dimorphic as an adaptive response to maintain physiological and behavioral distinctions between males and females throughout their lives [77]. Sex differences in the overall ecological structure and composition of the gut microbiome are extensively reported, both in healthy individuals and in those with various diseases [28, 29, 71, 78, 79]. In the present study, ten genera were exclusively present in females, whereas one genus was specific to males. Additionally, before exposure to SPS, the alpha diversity, which assesses differences in within-subjects diversity, was significantly higher in females than in males. This pattern remained consistent even after SPS exposure. In fact, higher alpha diversity in females is a recurring observation reported in preclinical and clinical studies [80–82].

In addition to the within-subjects diversity, the overall microbial composition, as measured by beta diversity, also differed between the sexes. While the dissimilarity of the microbial ecosystem remained stable between male and female unstressed controls, the SPS-exposed groups displayed interesting shifts. Before exposure to SPS, the SPS-S subgroup displayed a clear sex separation similar to that of the unstressed controls, whereas no separation was observed in animals that subsequently became SPS-resilient, suggesting an overall shared similarity in composition at baseline between SPS-R males and females. Following exposure to SPS, a clear separation emerged in the beta diversity of the SPS-R subgroups, whereas that present in the SPS-S subgroups disappeared. This implied that the gut microbiota of resilient males and females responded differently to the stressor and underwent unique shifts in composition to withstand its effects, whereas in the SPS-S subgroups, the SPS stressor elicited a more uniform response.

While the overall microbial diversity and structure seemed to be comparable in the SPS-R subgroups before SPS and in the SPS-S subgroups following exposure to SPS, this does not necessarily mean that the specific microbial taxa and their functional capabilities are identical. In fact, differences in specific microbial genera and their metabolites can have cascading effects on host physiology and influence various pathways involved in the stress response, without causing apparent or overall changes in beta diversity [83–87]. In the current study, before exposure to SPS, the relative abundance of Lactobacillus was significantly higher, and the abundance of Lachnospiraceae_Incertae_Sedis was lower in SPS-R males than in the SPS-S subgroup, whereas the exact opposite was observed in females. Our data suggest that differential expression of the same microbial genera might represent a risk factor for predicting trauma susceptibility or resilience differently in males and females. For instance, pre-trauma low abundance of Lactobacillus and high abundance of Lachnospiraceae_Incertae_Sedis in females, but high abundance of Lactobacillus and low abundance of Lachnospiraceae_Incertae_Sedis in males, may contribute beneficially to the host’s ability to withstand SPS-induced maladaptive behavioral alterations. Hence, it is crucial to avoid interpreting compositional similarities within a population, without considering sex-specific interactions. In this context, even when exposed to the same microbial community, sex-specific responses, can significantly impact the treatment outcomes for restoring intestinal homeostasis [88]. Similarly, sex-specific interactions between microbiota and pharmaceutical compounds can contribute significantly to variations in drug response, effectiveness, and the occurrence of adverse reactions between males and females [89, 90]. Thus, by acknowledging and accounting for sex-specific differences in the gut microbiome, therapeutic approaches can be tailored to achieve more effective treatments. It is important to acknowledge that in this study and in many others, the analysis of microbial composition was conducted at the genus level. Genera can include various species and strains with distinct functionalities. This might also account to the contradictory results found in the literature regarding the beneficial or harmful effects of Lactobacillus [35, 91–103], Lachnospiraceae_Incertae_Sedis [104–110], and Barnesiella [111–113]. Therefore, it is crucial in the future to identify the specific species or strains of these genera that differ in both males and females.

Consistent with the compositional differences, microbial predictive functionality also differed between the sexes. Most of the microbial metabolic pathways of SPS-S males and females shifted in a similar manner after SPS, suggesting a potential shared foundation for stress vulnerability among the susceptible group. The observed shifts in these pathways may further suggest alterations in host–microbiome interactions, modifications in microbial cell signaling and communication, as well as dynamic adaptations of microbial communities in response to stress. Sex differences were also observed in the metabolism and neuroactive potential of the microbiota, in the targeted GBMs and GMMs, with the overall metabolism of different neurotransmitters being higher in females. This further supports the idea that gut microbial functionality is tailored to the needs of each sex.

SCFAs serve as key mediators in orchestrating host–microbiota interactions [114]. Beyond their recognized neuroprotective and anti-inflammatory attributes [115–119], SCFAs emerge as significant contributors to epigenetic regulation across diverse bodily organs like the colon, brain, liver, and white adipose tissues. Following exposure to SPS, sex-specific alterations in these microbial metabolites were apparent: while resilient males displayed elevated cecal acetate levels, susceptible females exhibited heightened branched-chain SCFAs. Notably, acetate, the predominant SCFA, assumes a vital role in histone modification through lysine acetylation, thereby conferring protection against stress-induced physiological and behavioral disruptions [119, 120]. While our assessment was directed solely on post-SPS acetate levels, the analysis of GBMs revealed that acetate synthesis I pathway of resilient males was higher even before SPS exposure. This suggests that acetate could play a pivotal role in ameliorating adverse SPS effects in males, potentially serving as a biomarker to distingushish susceptibility or resilience. In contrast, the branched-SCFAs, implicated primarily in intestinal inflammation, among susceptible females may potentially serve as indicative biomarkers for this subgroup [121]. It is also worth noting that the gut microbiome can impact brain function through regulation of BBB permeability [122]. In preclinical studies, suppression of claudin-5 protein induces anxiety and depressive-like behavior [123], whereas administration of microbial major SCFAs or the bacteria which produces them restores its expression [123].

Despite the recognized protective effects of acetate, no apparent group distinctions were observed among the females in cecal acetate levels nor in acetate synthesis pathways. Furthermore, SPS-R females exhibited lower acetate levels than their SPS-R male counterparts, hinting that acetate might hold a differing degree of significance in females compared to males. However, interestingly, the glutamate degradation I (leading to acetyl-coA and crotonyl-coA) and acetyl-coA to crotonyl-coA modules exhibited significant elevation in females compared to males, both before and after SPS. These modules could culminate in histone crotonylation, which is a recently identified post-translational modification (PTM) associated with microbial metabolic processes and adaptation to the environment [77, 124]. Host-wise, histone crotonylation, similar to acetylation, is mediated through SCFAs and is relatively abundant modification in the intestinal epithelium and the brain, with H3K18cr being the most prevalent histone crotonylation [125]. Although, the translation of these bacterial pathways to host outcomes might not be direct; however, the depletion of gut bacteria has an evident impact on global histone crotonylation levels within the gut [125]. Histone acetylation and crotonylation both induce epigenetic modifications that influence chromatin structure and gene expression [119, 126, 127]. Yet, their effects on nucleosome functionality and interactions with chromatin remodeling factors diverge [127]. For instance, within a cell-free assay, histone crotonylation surpassed acetylation in enhancing gene expression, by creating a unique binding platforms for YEATS domain-containing factors [128]. This further underscores the distinct metabolites required for orchestrating sex-specific epigenetic adaptations aimed at counteracting the effects of traumatic stress.

In conclusion, to the best of our knowledge, this is the first study to show pre-existing and trauma-induced sex differences in gut microbial composition, functionality, and metabolites. Our study sheds light on the intriguing sex-specific variations observed in the response to stress induced by SPS in male and female rats. Notably, these differences extend beyond behavioral and physiological aspects to encompass the gut microbiota and its metabolites. The identification of sex-specific microbial contributors to resilience and susceptibility highlights the necessity of developing tailored therapeutic strategies for stress-related disorders. Acknowledging the role of sex in stress responses and the gut–brain axis is essential for optimizing treatment approaches and accommodating individual differences between males and females.

### Perspectives and significance

In summary, this study's comprehensive approach underscores the interdisciplinary nature necessary for understanding stress-related disorders. Through the investigation of distinct stress responses in male and female rats, this research advances our understanding of sex-related biological influences on mental health outcomes, shedding light on mechanisms that may contribute to observed sex differences in stress-related disorders. The chosen stressor's ability to capture interindividual variations in trauma responses facilitates a deeper exploration of the factors and mechanisms underpinning stress resilience and susceptibility. This, in turn, offers insights that may help enhance resilience in humans. The identification of sex-specific alterations in microbial composition, functionality, and metabolites suggests potential avenues for the development of sex-specific therapeutic interventions for those vulnerable to stress-induced psychopathologies. Furthermore, the study aligns with the growing emphasis on precision medicine, which tailors interventions to individual idiosyncrasies. The knowledge gathered from this study holds the potential to not only deliver more precise, effective treatments adapted to each sex's unique needs, but also serve as source of potential biomarkers for early identification of stress-susceptible individuals. As a result, this study's implications span both basic science and clinical applications, offering promising insights into the intricate interactions between the microbiome, sex, stress, and mental health.

### Supplementary Information

**Additional file 1: Figure S1.** Distribution of total animals in each phase of the estrous cycle during SPS and behavioral tests. **Figure S2.** Sex differences in the gut microbial predictive functionality before and after SPS. **Figure S3.** Sex differences in the gut–brain module before and after SPS. **Figure S4.** Sex differences in the gut–metabolic module before SPS. **Figure S5.** Sex differences in the gut–metabolic module after SPS. **Figure S6.** Sex differences in cecal weight and cecal short chain fatty acids.

## Data Availability

Data will be made available on request.
