# Supplementary material for: Microbial composition, functionality, and stress resilience or susceptibility: unraveling sex-specific patterns
Source: Biol Sex Differ. 2024 Feb 26;15:20. doi: 10.1186/s13293-024-00590-7 (PMC10898170; doi:10.1186/s13293-024-00590-7)
Supplement: Supplementary file 1 — Additional file 1: Figure S1. Distribution of total animals in each phase of the estrous cycle during SPS and behavioral tests. Figure S2. Sex differences in the gut microbial predictive functionality before and after SPS. Figure S3. Sex differences in the gut–brain module before and after SPS. Figure S4. Sex differences in the gut–metabolic module before SPS. Figure S5. Sex differences in the gut–metabolic module after SPS. Figure S6. Sex differences in cecal weight and cecal short chain fatty acids. [file 13293_2024_590_MOESM1_ESM.pdf]

Additional File 1

Figures:

Figure S1*: Distribution of total animals in each phase of the estrous cycle during SPS and behavioral tests.*

Figure S2*: Sex differences in the gut microbial predictive functionality before and after SPS.*

Figure S3*: Sex differences in the gut-brain module before and after SPS.*

Figure S4*: Sex differences in the gut-metabolic module before SPS.*

Figure S5*: Sex differences in the gut-metabolic module after SPS.*

Figure S6*: Sex differences in cecal weight and cecal short chain fatty acids.*

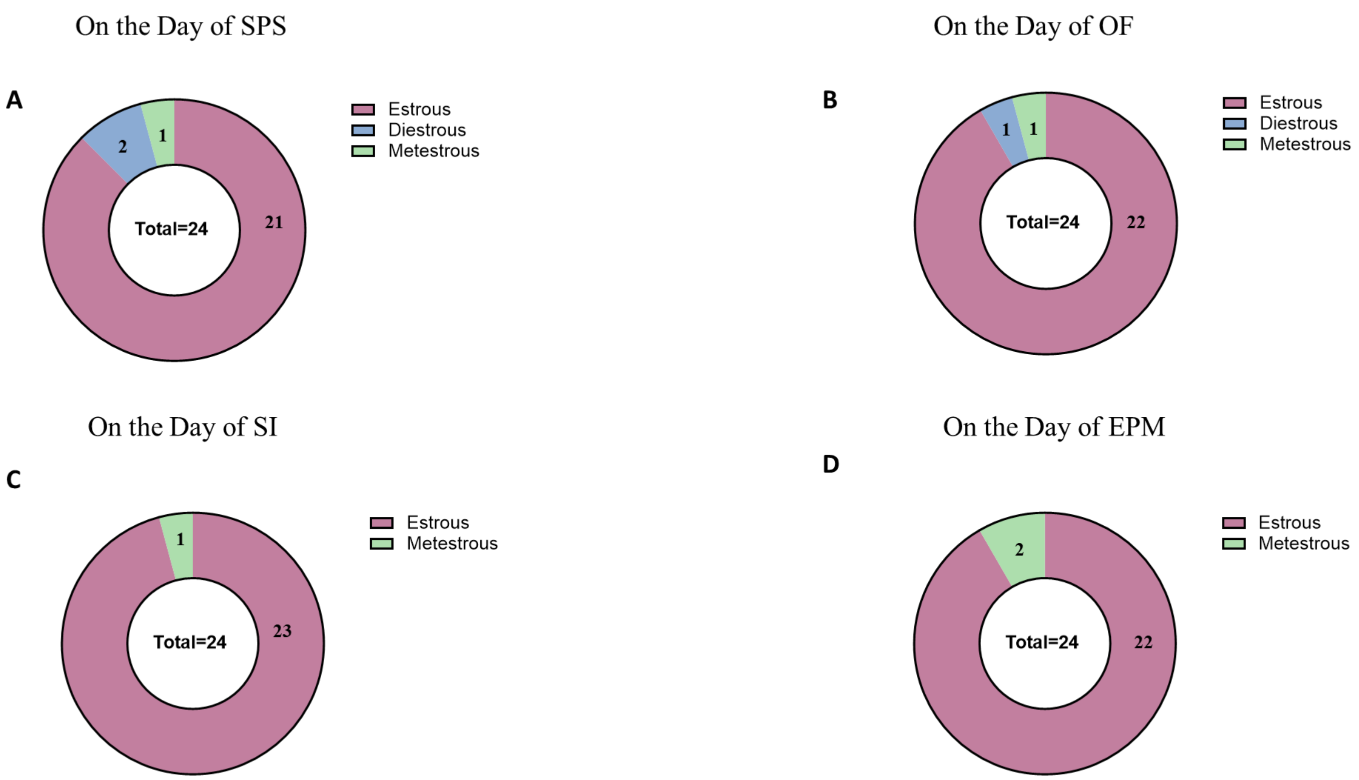

Figure S1: Distribution of total female rats in each phase of the estrous cycle during SPS and behavioral tests. **A**: On the days of SPS, **B**: On the day of OF, **C**: on the day of SI, **D**: On the day of EPM.

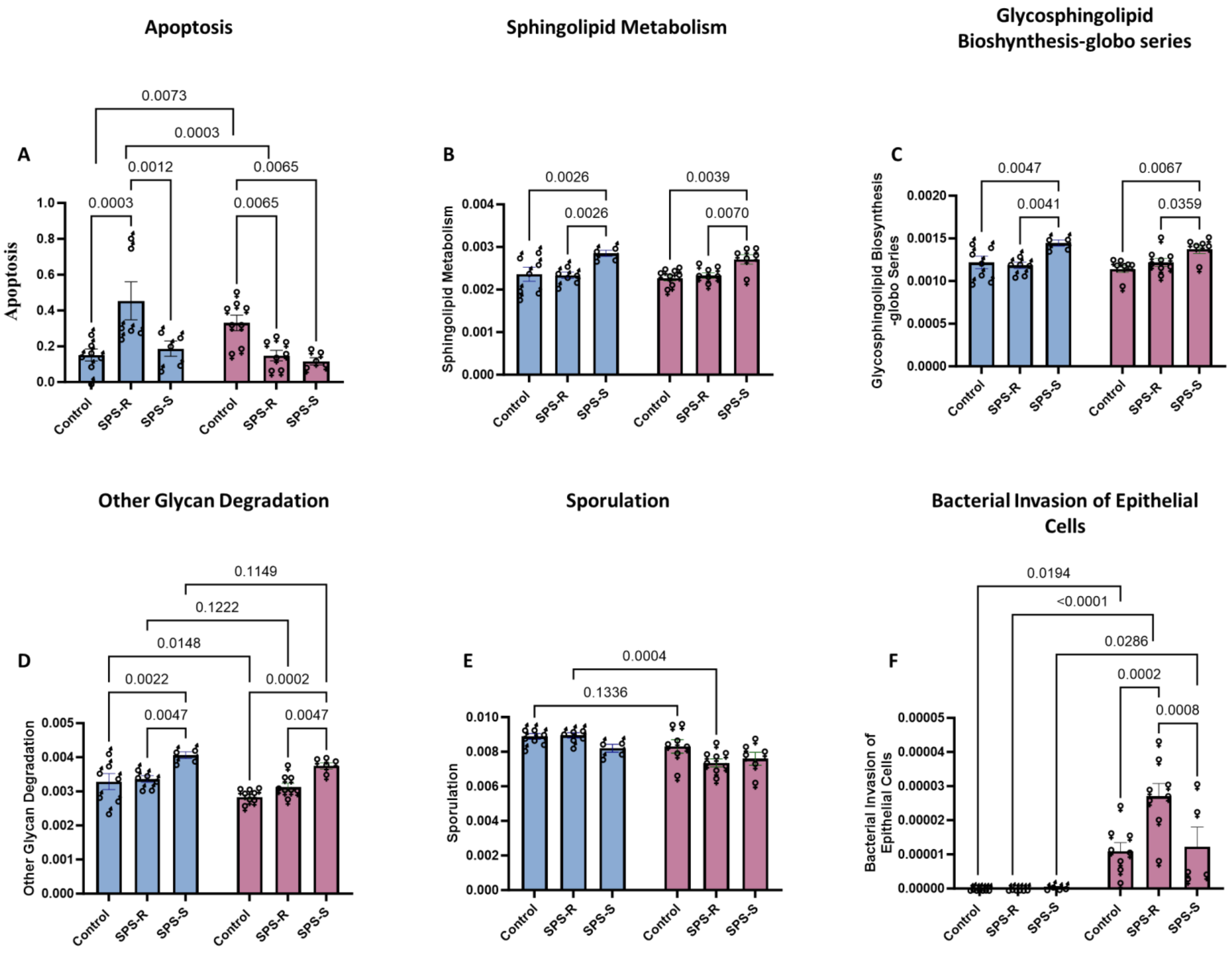

Figure S2: Sex differences in the gut microbial predictive functionality before and after SPS.

Before SPS exposure **A**: Pathways in Apoptosis, *Following SPS exposure* **B**: Sphingolipid metabolism, **C**: Glycosphingolipid Biosynthesis-globo series, **D**: Other glycan degradation, **E**: Sporulation, **F**: Bacterial invasion of epithelial cells. Due to technical difficulties stool samples were not collected from every single animal. Each symbol represents the value for an individual animal.

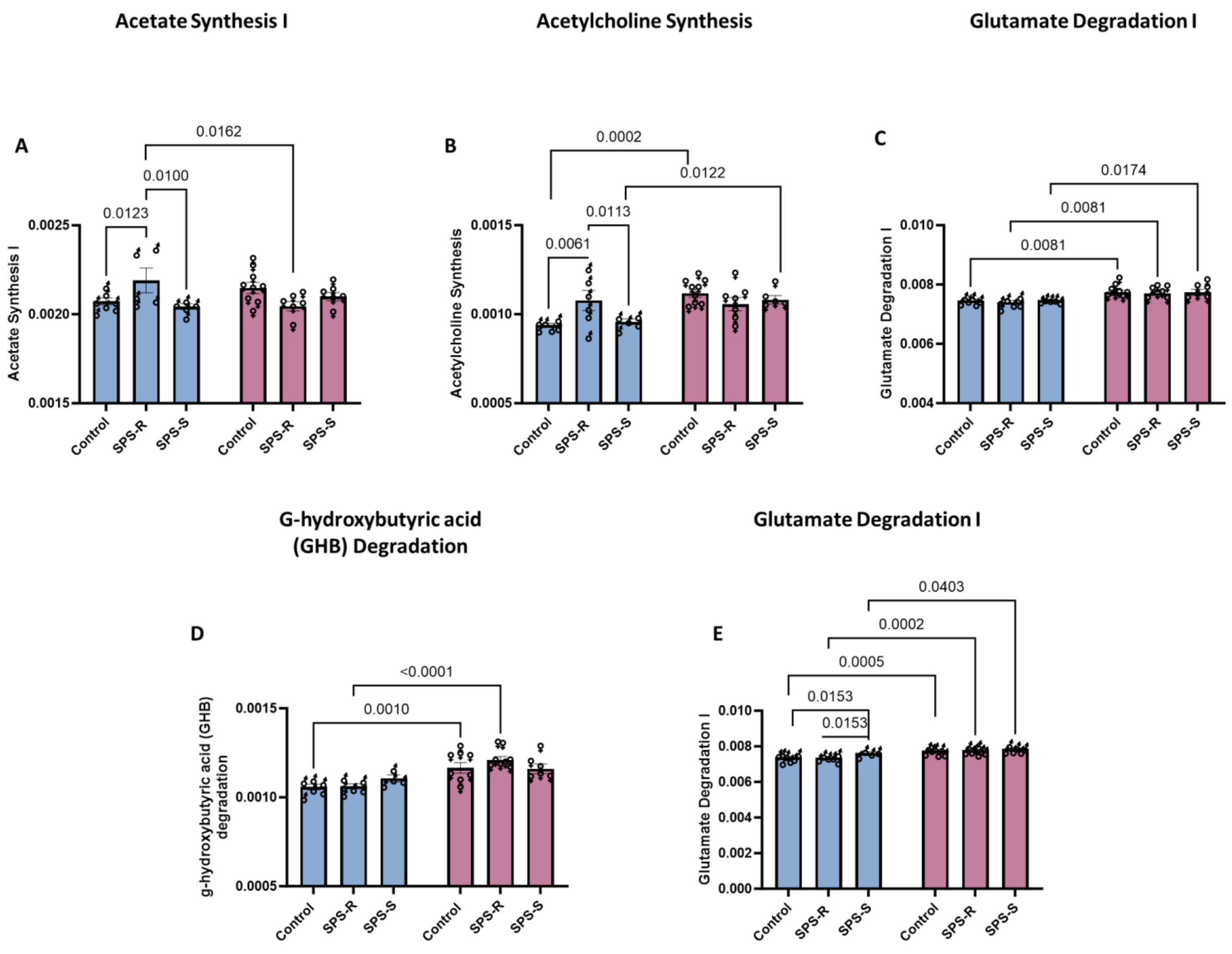

Figure S3: Sex differences in the gut-brain module before and after SPS

Before SPS exposure **A:** Acetate synthesis I**, B:** Acetylcholine synthesis, **C:** Glutamate degradation I, *Following SPS exposure* **D:** g-hydroxybutyric acid (GHB) degradation**, E:** Glutamate degradation I**.** Due to technical difficulties stool samples were not collected from every single animal. Each symbol represents the value for an individual animal.

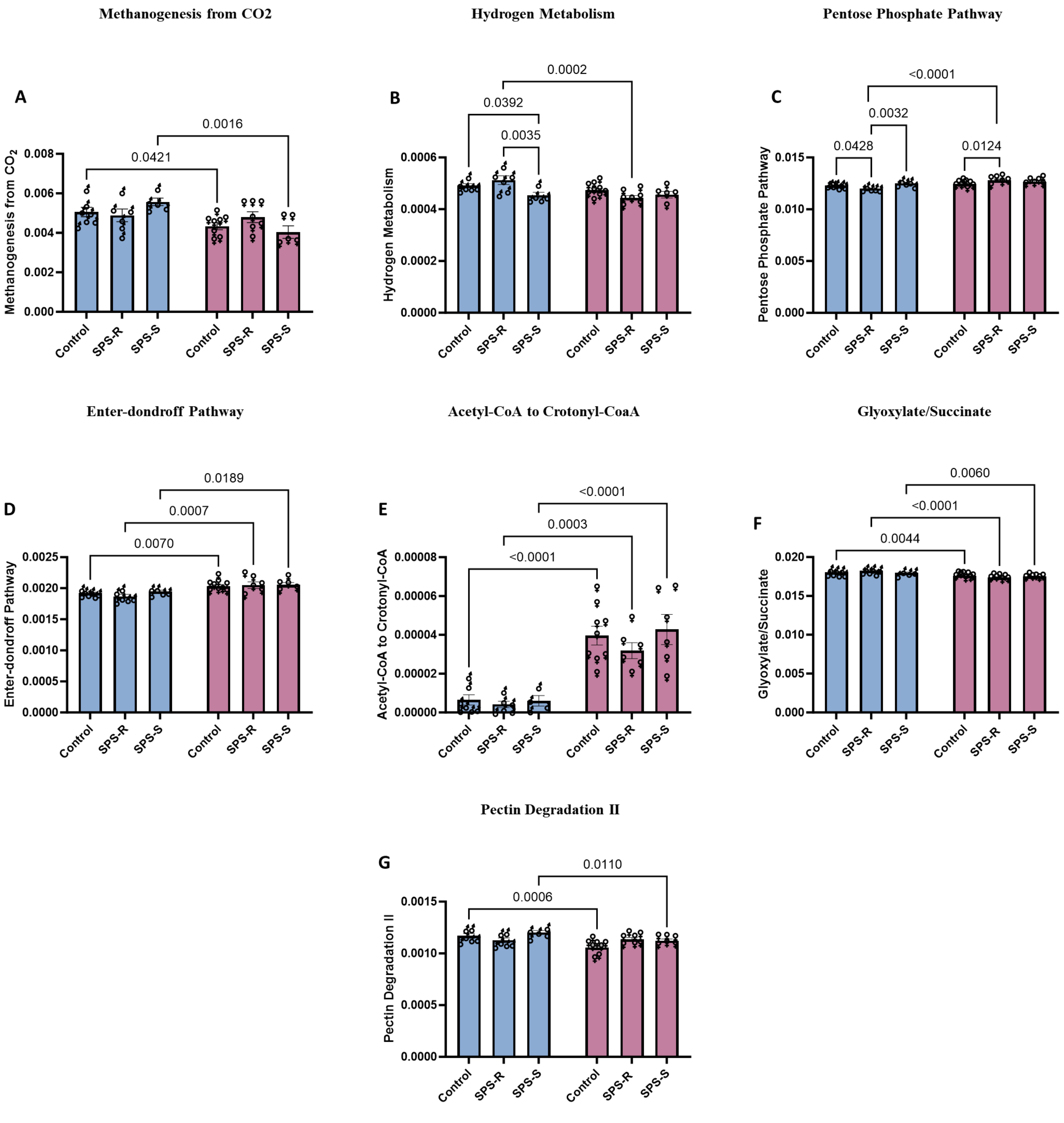

Figure S4: Sex differences in the gut-metabolic module before SPS

Before SPS exposure **A:** Methanogenesis from CO_2**, B:** Hydrogen metabolism, **C:** Pentose phosphate pathway, **D:** Enter-dondroff pathway**, E:** Acetyl-CoA to Crotonyl-CoA, **F**: Glyoxylate/Succinate, **G**: Pectin Degradation II. Due to technical difficulties stool samples were not collected from every single animal. Each symbol represents the value for an individual animal.

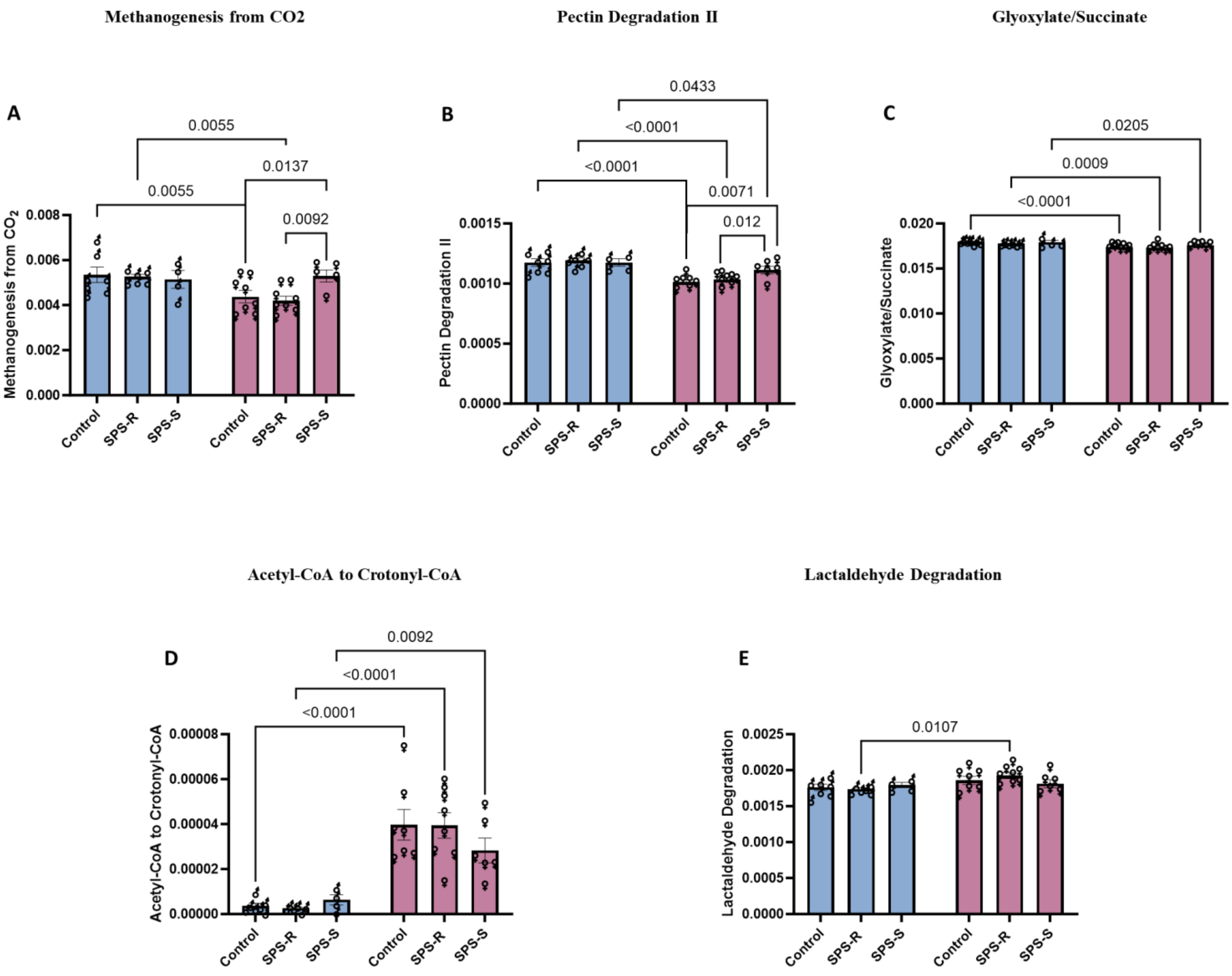

Figure S5: Sex differences in the gut-metabolic module after SPS

A: Methanogenesis from CO_2, **B:** Pectin Degradation II , **C:** Glyoxylate/Succinate**, D:** Acetyl-CoA to Crotonyl-CoA**, E:** Lactaldehyde degradation. Due to technical difficulties stool samples

were not collected from every single animal. Each symbol represents the value for an individual animal.

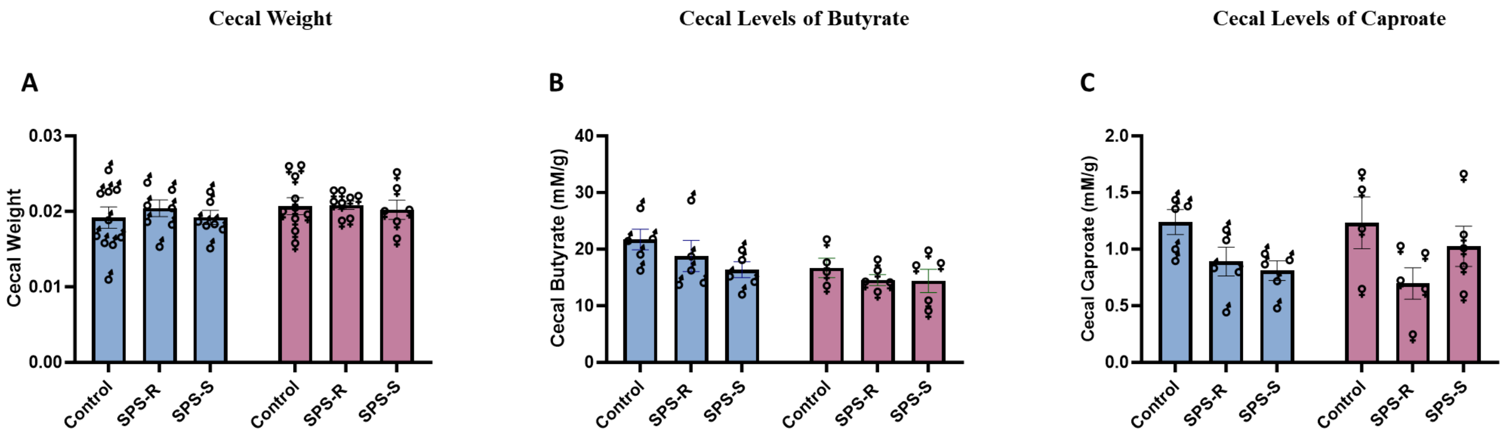

Figure S6: Sex differences in cecal weight and cecal short chain fatty acids.

A: Cecal weight normalized to body weight on the day of dissection**, B:** Levels of cecal butyrate**,** **C:** Levels of cecal caproate**.**
